# The mechanism study of targeting DPP4 in regulating ferroptosis and its influence on endometrial receptivity in PCOS

**DOI:** 10.1186/s13293-025-00786-5

**Published:** 2025-12-23

**Authors:** Jian Zhang, Ruifang Wang, Xiuli Tian, Nan Ding, Yajun He, Kexin Wang, Chengbin Tao, Jiao Cai, Qiliang Jian, Fang Wang

**Affiliations:** 1 Department of Reproductive Medicine, Lanzhou University Second Hospital, Lanzhou, 730030 China; 2 Department of Reproductive Medicine, Shanxi Provincial People’s Hospital, Taiyuan, 030012 China; 3 Department of Obstetrics and Gynecology, The First Affiliated Hospital, and College of Clinical Medicine of Henan University of Science and Technology, Luoyang, 471003 China

**Keywords:** Polycystic ovary syndrome, Endometrial receptivity, Ferroptosis, Dipeptidyl Peptidase 4, Endometrial stromal cells

## Abstract

**Background:**

Polycystic ovary syndrome (PCOS) impairs endometrial receptivity, contributing to reproductive dysfunction. Our previous work identified ferroptosis-related dipeptidyl Peptidase 4 (DPP4) as a key regulator of endometrial receptivity in PCOS, though its mechanism remained unclear.

**Objective:**

We aimed to explore the regulatory mechanism of DPP4 in the occurrence and tolerance of endometrial ferroptosis in PCOS.

**Methods:**

Using high dose (HD) DHEA-induced rats and hormone-treated (E2 and HD DHEA) telomerase-immortalized human endometrial stromal cells (T-HESCs), we investigated DPP4’s role in endometrial ferroptosis and receptivity. We evaluated the correlation of specific endometrial marker expression levels with reproductive outcomes.

**Results:**

Phenotypic assessments revealed elevated endometrial Fe^2+^ accumulation, antioxidant dysfunction, mitochondrial damage, and enhanced estrogen/androgen receptor expression in PCOS models. DPP4 inhibition via sitagliptin improved decidualization responses and receptivity markers in rats prior to pregnancy. In T-HESCs, downregulated DPP4 could suppress hormone receptor expression and ferroptosis markers. Functional validation using BeWo spheroid implantation assays demonstrated restored endometrial receptivity following DPP4 intervention. Mechanistically, DPP4-driven ferroptosis exacerbated PCOS-associated endometrial dysfunction, while its suppression would enhance stromal cell decidualization capacity and implantation potential. Consistent with these findings, evaluation of endometrial specimens from PCOS patients confirmed a marked reversal of ferroptosis-related markers following sitagliptin intervention, which was further associated with significantly improved reproductive outcomes, including higher clinical pregnancy and live birth rates.

**Conclusion:**

Reducing DPP4 expression not only inhibited ferroptosis but also improved the PCOS phenotype of the endometrium, ultimately influencing changes in endometrial receptivity, and indicating the ferroptosis-related protein DPP4 as a promising therapeutic target.

**Supplementary Information:**

The online version contains supplementary material available at 10.1186/s13293-025-00786-5.

## Plain language summary

Polycystic ovary syndrome (PCOS), a prevalent reproductive endocrine and metabolic disorder among women of childbearing age, poses significant clinical challenges due to its high prevalence and substantial health impacts. Emerging evidence suggests that impaired endometrial receptivity constitutes a critical pathological factor contributing to reproductive dysfunction in PCOS. Our preliminary investigations identified dipeptidyl peptidase-4 (DPP4), a ferroptosis-related protein, as a key regulator of endometrial receptivity and reproductive outcomes in PCOS, although its precise mechanistic role remains unclear. This study systematically investigates the mechanistic involvement of DPP4 in endometrial ferroptosis and receptivity impairment in PCOS. Utilizing a comprehensive experimental approach, we employed both a high dose dehydroepiandrosterone (HD DHEA)-induced PCOS rat model and telomerase-immortalized human endometrial stromal cells (T-HESCs) treated with estrogen (E2) and DHEA to explore DPP4’s role. Our findings reveal that PCOS rats exhibit: significant endometrial Fe^2^⁺ accumulation, compromised antioxidant defenses, mitochondrial dysfunction, and altered estrogen/androgen receptor expression profiles. Collectively, our results establish that targeted DPP4 inhibition not only mitigates ferroptosis but also ameliorates PCOS-associated endometrial dysfunction, ultimately normalizing receptivity parameters. These findings position DPP4 inhibition as a promising therapeutic strategy for PCOS-related fertility challenges, offering new mechanistic insights and potential clinical applications.

**Supplementary Information:**

The online version contains supplementary material available at 10.1186/s13293-025-00786-5.

## Highlights

Ferroptosis may represent a novel mechanism underlying impaired endometrial receptivity in PCOS, with DPP4 identified as a key protein mediating both endometrial dysfunction and ferroptosis in PCOS. Inhibition of ferroptosis shows potential for improving endometrial receptivity.

In vitro studies using HD DHEA-induced endometrial stromal cells demonstrate the occurrence of ferroptosis, while pharmacological inhibition of this process exerts beneficial effects on stromal cell decidualization.

Downregulation of DPP4 expression in HD DHEA-induced endometrial stromal cells effectively reduces ferroptosis incidence.

Targeted modulation of DPP4 protein expression emerges as a promising therapeutic strategy for improving endometrial receptivity in PCOS.

The DPP4 inhibitor sitagliptin demonstrates remarkable efficacy in animal studies, not only increasing embryo implantation rates in HD DHEA-induced rats but also significantly improving endometrial receptivity, providing substantial evidence for its potential clinical translation in PCOS-related infertility treatment.

Polycystic ovary syndrome (PCOS), a prevalent reproductive endocrine and metabolic disorder among women of childbearing age, poses significant clinical challenges due to its high prevalence and substantial health impacts. Emerging evidence suggests that impaired endometrial receptivity constitutes a critical pathological factor contributing to reproductive dysfunction in PCOS. Our preliminary investigations identified dipeptidyl peptidase-4 (DPP4), a ferroptosis-related protein, as a key regulator of endometrial receptivity and reproductive outcomes in PCOS, although its precise mechanistic role remains unclear. This study systematically investigates the mechanistic involvement of DPP4 in endometrial ferroptosis and receptivity impairment in PCOS. Utilizing a comprehensive experimental approach, we employed both a high dose dehydroepiandrosterone (HD DHEA)-induced PCOS rat model and telomerase-immortalized human endometrial stromal cells (T-HESCs) treated with estrogen (E2) and DHEA to explore DPP4’s role. Our findings reveal that PCOS rats exhibit: significant endometrial Fe^2^⁺ accumulation, compromised antioxidant defenses, mitochondrial dysfunction, and altered estrogen/androgen receptor expression profiles. Collectively, our results establish that targeted DPP4 inhibition not only mitigates ferroptosis but also ameliorates PCOS-associated endometrial dysfunction, ultimately normalizing receptivity parameters. These findings position DPP4 inhibition as a promising therapeutic strategy for PCOS-related fertility challenges, offering new mechanistic insights and potential clinical applications.

**Supplementary Information:**

The online version contains supplementary material available at 10.1186/s13293-025-00786-5.

## Introduction

The current knowledge of endometrial receptivity in polycystic ovary syndrome (PCOS) patients is continually expanding as new studies become available. Endometrial receptivity refers to the ability of the endometrium to allow normal embryo implantation, and it is a crucial factor in the establishment of a successful pregnancy [1]. Initially, the majority of PCOS research focused on promoting ovulation and improving oocyte quality. However, recent efforts have focused more on the importance of endometrial receptivity for successful pregnancy [2–6]. Furthermore, hyperandrogenism, inflammation, insulin resistance, and obesity have been implicated in disrupting normal endometrial functions [7–10]. Some studies have explored the molecular mechanism of endometrial receptivity defects in patients with PCOS [11, 12]. Although impaired endometrial receptivity is a significant cause of infertility in PCOS, it has received less research attention compared to ovulatory dysfunction. Growing evidence indicates that endometrial dysfunction contributes to recurrent implantation failure and miscarriage in women with PCOS [7]. A study indicated that treated PCOS patients who achieved ovulation presented markedly lower clinical pregnancy and live birth rates than non-PCOS individuals did, implying that compromised endometrial receptivity was a pivotal component influencing their reproductive function [13]. Moreover, challenges in the decidualization process in patients with PCOS could further impair endometrial receptivity, adversely affecting reproductive results [14, 15]. Consequently, augmenting endometrial receptivity and promoting normal decidualization in people with PCOS may be essential strategies for increasing conception rates.

Recent studies have shown that mitochondrial dysfunction and elevated oxidative stress are implicated in the pathogenesis of PCOS and its associated comorbidities. The increased formation of reactive oxygen species (ROS) may cause oxidative stress, which can damage mitochondrial DNA (mtDNA), proteins, and lipids [12]. Ferroptosis is an iron-dependent form of regulated cell death characterized by intracellular Fe^2^⁺ accumulation, mitochondrial dysfunction and elevated oxidative stress [16, 17]. Extracellular Fe^3^⁺ enters cells via transferrin receptor 1 (TFR1) and is reduced to Fe^2^⁺, which drives lipid oxidation through the Fenton reaction. This process depletes reduced glutathione (GSH) and downregulates glutathione peroxidase 4 (GPX4)—a hallmark enzyme of ferroptosis suppression—thereby leading to massive reactive oxygen species (ROS) production and ultimately triggering ferroptosis [16]. In PCOS, oxidative stress and mitochondrial dysfunction may trigger ferroptosis [18–21]. Our prior proteomic study identified evidence of ferroptosis in the endometrium of PCOS patients. This finding suggested that ferroptosis could be a mechanism contributing to impaired endometrial receptivity and recurrent pregnancy loss in this population. We identified the ferroptosis-related protein dipeptidyl peptidase 4 (DPP4) as a potential key regulator of endometrial receptivity in PCOS [18]. Although existing studies had indicated a link between DPP4 and ferroptosis, a comprehensive understanding of how DPP4 affected the ferroptosis process and endometrial function was lacking. For this investigation, we employed two strategies to modulate DPP4: lentiviral transfection and pharmacological inhibition. Sitagliptin is a widely prescribed and effective DPP4 inhibitor for managing type 2 diabetes [22, 23]. To determine if DPP4 inhibition ameliorates endometrial receptivity defects in PCOS by modulating ferroptosis, we utilized sitagliptin to investigate the underlying mechanisms. Sitagliptin is commonly used to treat type 2 diabetes and has demonstrated good clinical efficacy [22–25]. The literature also reported attempts to utilize sitagliptin to improve ovarian function and insulin resistance in PCOS [26, 27]. However, the role of DPP4 in regulating endometrial receptivity remains unexplored. An in-depth investigation into the role of DPP4 regulating ferroptosis would enhance our understanding of improvements in the status of endometrial stromal cells in PCOS.

Therefore, this study aimed to investigate the roles of DPP4 and ferroptosis in the PCOS endometrium and to elucidate the mechanism by which reduced DPP4 inhibited ferroptosis to improve endometrial receptivity. A deep understanding of these processes is crucial for developing new therapeutic strategies.

## Materials and methods

### Clinical samples collection

The endometrium samples and patients’ clinical information were collected from 33 women with PCOS before and after treatment with sitagliptin at the Reproductive Center of Lanzhou University Second Hospital. PCOS was diagnosed according to the Rotterdam criteria. These patients were required to meet all the following conditions: no use of relevant medications within 3 months before enrollment, voluntary participation with good compliance, married female status, and normal sperm from the spouse. Exclusion criteria included: use of hormonal agents or drugs affecting insulin secretion within the past 3 months, and presence of cardiovascular, hepatic, renal, or hematopoietic system diseases. The control group consisted of non-PCOS women with regular menstrual cycles, normal ovarian morphology, and a history of successful pregnancy. All participants provided informed consent, and the study protocol was approved by the Ethics Committee of Lanzhou University Second Hospital (2024A-107).

Endometrial tissue samples were collected using a Pipelle endometrial aspirator. One portion of the samples was rinsed with phosphate-buffered saline (PBS) and stored at − 80 °C for future use, while the other portion underwent pathological examination. Only endometrial specimens obtained during the proliferative phase were selected for analysis. Reproductive outcomes served as the key indicators for evaluating endometrial receptivity, including live birth and adverse fertility outcomes (such as abortion and implantation failure).

### Cell culture and animals’ housing conditions

Telomerase-immortalized human endometrial stromal cells (T-HESCs, #CRL-4003) and BeWo cells (#CCL-98) were obtained from the American Type Culture Collection (ATCC, USA). The T-HESCs were cultured in DMEM/F12 (Thermo Fisher Scientific, Waltham, MA, USA) medium supplemented with 10% fetal bovine serum (FBS, Excell Bio, Suzhou, Jiangsu, China) in a humidified incubator at 37 °C with 5% CO_2_.

The Specific Pathogen Free grade (SPF-grade) Sprague–Dawley (SD) female rats (n = 51, aged 3 weeks) and male rats (n = 27, aged 8 weeks) were procured from the Experimental Animal Center at Lanzhou University (Lanzhou, Gansu, China). The rats were maintained under a controlled temperature of 20–22℃, 40–60% humidity, 12 h of light/dark cycle lighting conditions, and standard laboratory conditions with SPF-grade rodent feed and water.

### PCOS-like model rats and experiment design

**Fig. 1:**
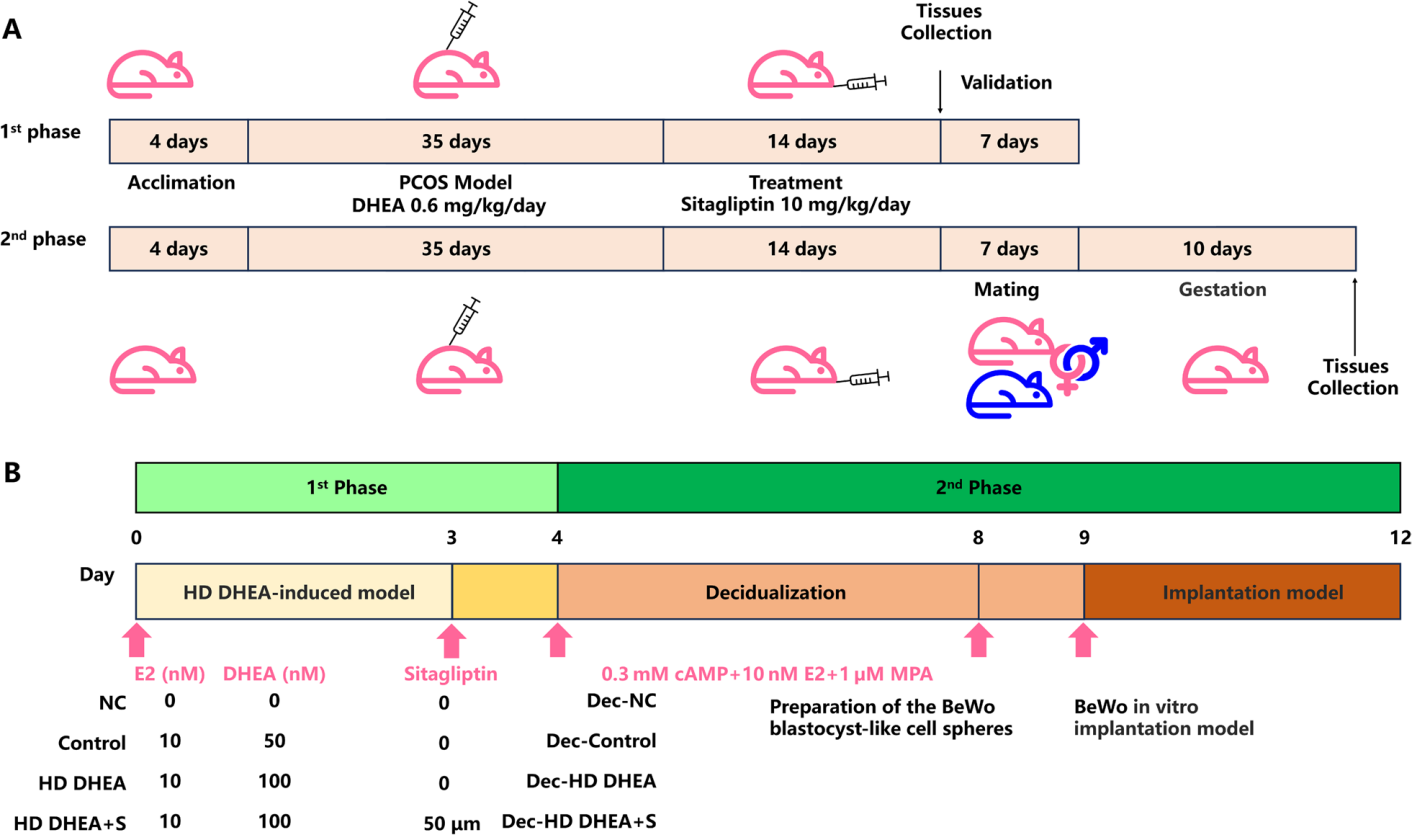
Schematic diagram of the experimental design**. A.** The schematic diagram of PCOS-like model rats and experiment design. **B.** The schematic diagram of the HD DHEA cell experiment

The establishment of the rat model was comparable to that of a previous description [28, 29]. Please refer to Fig. 1A for the experimental research in this section. To induce PCOS rats, the rats were subcutaneous injection of dehydroepiandrosterone (DHEA, Abmole, Wuhan, Hubei, China, suspended in soybean oil) 0.6 mg/kg/day into the back of rats for 35 days. In the rat model study, the groups were divided as follows: the negative control group (NC, no intervention), the Control group (administered with oil without DHEA via dorsal injection), and the PCOS group (administered with DHEA oil solution via dorsal injection).

In the first phase of the experiment, we will validate the model and select an appropriate dose of sitagliptin for the second phase of the experiment. The research design was shown in Fig. 1A. In the first phase, rats were treated with sitagliptin (Abmole, Wuhan, Hubei, China) at three different doses for two weeks—low (2 mg/kg), medium (10 mg/kg), and high (50 mg/kg), further elucidate the role of DPP4 inhibition in reducing ferroptosis and improving endometrial receptivity in PCOS. Ferrostatin-1 (FER-1, Abmole, Wuhan, Hubei, China) was a substance that blocks the process of ferroptosis and was used as a negative control group for effectively preventing ferroptosis from happening [30]. As well as ferristatin-1 at a dose of 1 mg/kg, each administered for two weeks via daily gavage. Upon completion of this phase, all rats were euthanized using 3% pentobarbital sodium (Jiehui Biotechnology, Beijing, China, 50 mg/kg), and serum, ovaries, and uterus samples were collected and either fixed in formalin or stored at −80 °C for future analysis [31].

The next stage of the experiment was carried out once the appropriate sitagliptin concentration group was chosen in the previous stage. In the next phase, a pregnant rat model was established to evaluate endometrial receptivity. Male and female rats were raised in a 3:2 ratio to achieve pregnancy. Mating was confirmed by the presence of a vaginal plug, which marked gestational day 0 (GD 0) [19]. The body weight of the rats was monitored daily. Three pregnant SD female rats were killed on the mornings of the GD 10 using an overdose of pentobarbital sodium (50 mg/kg) so that the uteri could be extracted and examined. The decidualization of the endometrium in rats occurs after conception, and the site for histological examination is the basal decidua under the placenta. In accordance with the referenced literature, the embryo absorptivity rate (EAR) was calculated using the formula EAR = Re/(Re + Le) to assess the rates of resorbed and viable embryos [32]. Here, Re represented the number of reabsorbed embryos, while Le denoted the number of viable embryos. The method for identifying the number of embryos in the uterus involved recognizing differences in size and color. Resorbed embryos, which were affected by ischemia, hemorrhage, and necrosis, were significantly smaller in size compared to normal embryos. Additionally, there was a distinct color difference between resorbed and normal embryos: resorbed embryos appear dark brown, while normal embryos are pink.

### Cell experiment design

In this study, we employed two approaches to target the DPP4 protein: lentiviral transfection and pharmacological inhibition. Specifically, we used sitagliptin, a well-known DPP4 inhibitor, as our primary pharmacological tool. Erastin (ERA, Abmole, Wuhan, Hubei, China) was a substance that triggers ferroptosis and acts as a positive control for stimulating the onset of ferroptosis [33]. The concentration of ferrostatin-1 (1 μM) and erastin (2.5 μM) was used in a previously reported methodology [34].

To investigate the effects of DPP4 and ferroptosis on the HD DHEA-induced model of T-HESCs, the cell research was divided into two phases. In the first phase, we will examine the interrelationships among DPP4, ferroptosis, and the PCOS phenotype using a HD DHEA-induced cell model. In the second phase, we plan to induce cell decidualization using in vitro BeWo blastocyst-like cell sphere implantation models, and assess endometrial receptivity. The cell research design is illustrated in Fig. 1B.

### HD DHEA-induced model induction condition in T-HESCs

For the induction of HD DHEA-induced in T-HESCs by using DHEA, briefly, cells were first grown to 50% confluency and then treated with DHEA (0, 10, 25, 50, 75, 100, 150, 200, and 300 nM) for 24 h, 48 h, and 72 h in complete medium (DMEM/F12 + 10%FBS), and then detected using the cell counting kit-8 kit (CCK-8, Abmole, Wuhan, Hubei, China). To evaluate the toxicity after DHEA and determine the appropriate dose of DHEA, cell viability was determined using the CCK-8 kit. It was generally believed that a 50% survival rate indicates that the damage effect can be determined [35]. According to the literature, in most cases, the serum testosterone levels in women with PCOS do not exceed twice the average level of normal women [36, 37]. Therefore, we first determined the appropriate concentration of DHEA for the PCOS model, and used half of that concentration as the DHEA level for normal women. Determine the appropriate concentration of Estradiol (E2, Abmole, Wuhan, Hubei, China) in E2 + DHEA using the CCK-8 kit. E2 concentration was screened in the range of 0, 5, 10, 20, 50, 75, and 100 nM. The appropriate concentration of sitagliptin was screened in the range of 0, 10, 25, 50, 75, 100, 150, 200, and 300 μM with the appropriate concentration of E2 + DHEA. The T-HESCs viability was evaluated to assess and ascertain the suitable dosage of DHEA and E2 using the CCK-8. Following our experiments, 100 nM DHEA and 10 nM E2 was used as HD DHEA-induced model induction condition. Furthermore, 50 nM DHEA and 10 nM E2 as control model (normal) induction condition (Supplementary Figs. 1 A, 1B, and 1 C). If at a certain dose, the cell proliferation/survival rate reaches a high level, and the signaling pathways we are concerned about are significantly activated, and the expression of related genes or proteins meets our expectations, then this dose can be considered the optimal dose. Appropriate concentrations of sitagliptin (50 μM) was determined by using the CCK-8 (Supplementary Fig. 1D).

### Lentiviral transduction

Lentivirus with overexpression RNA and small interfering RNA (siRNA) sequences of DPP4 targeting humans was purchased from HanBio (Shanghai, China). Transfections with overexpression RNA-DPP4 and siRNA-DPP4 were performed according to the manufacturer’s protocol. Briefly, T-HESCs were placed into a six-well cell culture plate and transfected with LipoFiter™ Liposomal Transfection Reagent. 400 μg/ml geneticin (G418, HanBio, Shanghai, China) was used to screen stable knockdown and overexpression transfection cell lines. The effective sequences were as follows:

Overexpression RNA- DPP4 sense:

5′- TACTAGAGGATCTATTTCCGGTGAATTCGCCACCATGAAGACACCGTG −3′,

Overexpression RNA- DPP4 antisense:

5′-GCACCGGAGCGATCGCAGATCCTTTTATTTGTCGTCATCATCCTTAT-3′,

siRNA: TTCTCCGAACGTGTCACGTAA.

shRNA- DPP4 top strand:

5′- GATCCGTTCTCCGAACGTGTCACGTAATTCAAGAGATTACGTGACACGTTCGGAGAATTTTTTC− 3′,

shRNA-DPP4 bottom strand:

5′- AATTGAAAAAATTCTCCGAACGTGTCACGTAATCTCTTGAATTACGTGACACGTTC GGAGAACG − 3′.

### T-HESCs decidualization induction condition

To execute the decidualization induction condition, we used a previously reported methodology [38, 39]. T-HESCs were cultured in DMEM/F12 with 2% FBS, 0.3 mM cyclic adenosine monophosphate salt (cAMP, Abmole, Wuhan, Hubei, China), 10 nM E2, and 1 µM medroxyprogesterone (MPA, Abmole, Wuhan, Hubei, China). The induction medium was replaced by 1/2 volume every other day, continuously for 5 days.

### Preparation of BeWo blastocyst-like cell spheres and in vitro implantation model

To prepare BeWo blastocyst-like cell spheres, a 10 mg/mL Poly-HEMA solution (#P3932, Sigma, St. Louis, MO, USA) was first prepared using 37 °C anhydrous ethanol. In a biosafety cabinet, 1 mL of this solution was added to a 60 mm cell culture dish to fully cover the bottom, and then incubated in a cell culture incubator for 5 min. After removing the liquid and allowing the dish to dry, it was rinsed with PBS five times and irradiated with UV light for 15 min. BeWo cells were digested and resuspended to a concentration of 80–100 × 10^2^ cells/μL, and 20 μL of the cell suspension was spotted onto the dish using the hanging drop technique, followed by incubation for 2–3 h. Once cell clusters were confirmed under a microscope, 4 mL of culture medium was added. After 48 h, the cell clusters were filtered through a 100 μm cell strainer to obtain BeWo blastocyst-like cell spheres with a diameter of 100–150 μm.

For the decidualization of T-HESCs, cells were divided into four groups: blank (Dec-NC), control (Dec-Control), HD DHEA (Dec-HD DHEA), and PCOS with sitagliptin (Dec-HD DHEA + S). Five different culture media were prepared: Medium A (E2 + 1/2 DHEA), Medium B (E2 + DHEA), Medium C (normal complete medium), Medium D (sitagliptin), and Medium E (decidualization induction medium). Cells were plated one day prior to treatment at a density of 20–30%. The treatment schedule was as follows: Medium A and B on Day 0 and Day 2, Medium C and D on Day 3, and Medium E for all groups from Day 4 for 5 days. On Day 7, BeWo blastocyst-like cell spheres were prepared, and on Day 9, the in vitro implantation model was established [40–42]. Implantation spread and invasion ratios were observed and analyzed using ImageJ at 12 and 72 h post-implantation, with data normalized to the Dec-NC group, the calculation formula is as follows: $$\text{The ratio of invasion area }\left(\text{\%}\right)=$$$$\frac{\left({\text{Area}}_{\text{X group }72\text{h}}-{\text{Area}}_{\text{X group }12\text{h}}\right)}{\text{Mean}\left({\text{Area}}_{\text{Dec}-\text{NC }72\text{h}}-{\text{Area}}_{\text{Dec}-\text{NC }12\text{h}}\right)}\times 100 \%$$, (Fig. 1B).

### Relevant evaluation of molecular indicators

The PCOS phenotypes of T-HESCs and rat models were estimated by using western blotting of sex hormone receptor-associated protein levels of estrogen receptor (ER) and androgen receptor (AR) [43]. The decidualization phenotypes of T-HESCs and pregnancy rat model were estimated by using western blotting of sex hormone receptor-associated protein levels of ER, progesterone receptor (PR), and prolactin (PRL). The evaluation index of endometrial receptivity was homeobox A10 (HOXA10) and insulin-like growth factor binding protein 1 (IGFBP1) [44, 45].

This research will evaluate ferroptosis based on four aspects: Fe^2+^ concentration, oxidative stress state, mitochondrial condition, and expression of ferroptosis-associated proteins. The assessment of Fe^2+^ content included the use of Lillie’s staining technique and a Fe^2+^ content assay. The assessment of oxidative stress state included the examination of reactive oxygen species (ROS), malondialdehyde (MDA), and reduced GSH levels. The assessment of mitochondrial condition included the detection of mitochondrial membrane potential using JC-1 and the use of Transmission Electron Microscopy (TEM) for detection. The protein markers used were GPX4 and DPP4 [18].

### H&E staining examination

Ovary tissues were stained using the H&E staining kit (Solarbio, Beijing, China) according to the standard protocol. Each group of paraffin Sects. (4 µm) was deparaffinized and rehydrated before staining. Paraffin sections were prepared from the tissues. Subsequently, the sections were incubated in the hematoxylin and eosin staining solution. The stained ovary tissue sections were then observed and captured using an optical microscope (BX53 + DP74, Olympus, Tokyo, Japan).

### Lillie’s staining examination

Lillie’s staining was determined for cells and tissues using the Lillie’s staining kit (Solarbio, Beijing, China). The cells were then fixed with 10% formalin (pH 7.4) at room temperature for 15 min. Routine paraffin sections were prepared from tissues. Next, the cells and tissues were incubated in freshly prepared Lillie’s staining solution for 30 min. Cells and tissues were taken using an optical microscope.

### Measuring Fe^2+^, LPO, MDA, and GSH concentrations

The excessive accumulation of lipid peroxides (LPO) is a hallmark biomarker of ferroptosis. Malondialdehyde (MDA), a terminal product of LPO degradation, serves as a proxy indicator for assessing LPO levels. Conversely, reduced GSH directly neutralizes LPO. Its depletion consequently compromises cellular capacity to eliminate LPO, thereby accelerating ferroptosis occurrence [46]. Measuring Fe^2+^ (Elabscience, Wuhan, Hubei, China), LPO (Elabscience, Wuhan, Hubei, China), MDA (Elabscience, Wuhan, Hubei, China), and GSH (Elabscience, Wuhan, Hubei, China) concentrations in cells and tissues were detected using as per the manufacturer’s instructions.

### Protein extraction and western blotting

T-HESCs protein extract and snap-frozen rat endometrium tissues were used for western blotting. T-HESCs were lysed on ice for 30 min using SDS lysis buffer (Beyotime, Shanghai, China) supplemented with proteasome inhibitors (Servicebio, Wuhan, Hubei, China). For endometrium tissue, 1 mg of tissue was homogenized with 1 ml of SDS lysate using liquid nitrogen. The supernatants were obtained by centrifuging the lysate at 3000 rpm for 10 min at 4 °C and then incubating on ice for 20 min. The supernatant protein levels were assessed using an ultramicro-ultraviolet visible spectrophotometer (DENOVIX, Wilmington, DE, USA) according to the manufacturer’s instructions after centrifuging the lysates (12,000 rpm, 20 min, 4 °C). Approximately 30 µg of protein samples were loaded in each lane. SDS-PAGE separated proteins before they were transferred to PVDF membranes and blocked with 10% skim milk for 1 h. The transferred PVDF membranes were incubated with primary antibodies overnight at 4 °C and with HRP-conjugated secondary antibodies for 1 h at room temperature. β-actin was used as a loading control. The blots were detected and visualized using a fully automated chemiluminescence image processing system (Tanon, Shanghai, China) with an electrochemical luminescence reagent (ECL, Biosharp, Hefei, Anhui, China). For quantification and to ensure consistency across blots, the expression levels of the target proteins were normalized to the mean value of the control group on each blot. Subsequently, the normalized values were statistically compared to evaluate the effects of the treatment groups, as previously described.

The following antibodies were used: AR, ER, PR, GPX4, HRP-conjugated peroxidase secondary antibody were purchased from Proteintech, Wuhan, Hubei, China. PRL, IGFBP1, and HOXA10 were purchased from Affinity Bio, Changzhou, Jiangsu, China. DPP4 was purchased from ImmunoWay, Suzhou, Jiangsu, China. The primary antibodies were all diluted at a ratio of 1:1000, and the secondary antibody was diluted at a ratio of 1:5000. The primary antibodies were all rabbit host polyclonal antibodies, while the secondary antibody was the goat anti-rabbit HRP-conjugated antibody.

### Cell immunohistochemistry

Because of fluorescently labeled lentiviruses were used for transfection in the cell experiments, immunohistochemistry was employed for protein staining in the subsequent experiments. Cells were fixed with 10% formalin (pH 7.4) at room temperature for 30 min, permeabilized with 0.1% Triton X-100 (Beyotime, Shanghai, China) for 15 min, and blocked with quick blocking liquid (Beyotime, Shanghai, China) for 20 min. Then, the sections were incubated with the primary antibodies at 4 °C overnight (for over 10 h). The next day, the cells were incubated with an HRP-conjugated secondary antibody, followed by DAB (Servicebio, Wuhan, Hubei, China) chromogenic detection. The stained cells were observed under an optical microscope(BX53 + DP74, Olympus, Tokyo, Japan). To further determine the expression levels of relative proteins in the cells, the semi-quantitative analysis was conducted by calculating the mean optical density (MOD) using Image Pro Plus v. 6.0 software (Media Cybernetics, Rockville, USA). Photographs were taken at a magnification of × 40 under consistent exposure conditions, with six random images captured. The integrated optical density (IOD) was measured for each image. The mean optical density (MOD) was then calculated as follows: for images without blank areas, MOD = IOD/picture area; for images with blank areas, MOD = IOD/(picture area – blank area). The MOD value for each slice was determined as the average of the MOD values from the five randomly selected fields.

### Tyramide signal amplification (TSA) immunofluorescence

TSA Immunofluorescence staining was used to observe the expression of different target proteins in rat uterus tissues. Each group of paraffin Sects. (4 µm) was deparaffinized and rehydrated before staining. Then, the antigen in the slices was repaired using sodium citrate buffer (pH = 6.0) at 100℃. Subsequently, we quenched the tissue autofluorescence using Reagent B (Servicebio, Wuhan, Hubei, China), and incubated the slices with the primary antibody at 4 °C overnight (for more than 10 h). The next day, the primary antibodies were washed away, and then the slices were incubated with HRP-conjugated secondary antibody for 1 h. After that, the slices were incubated with TSA working fluid for 10 min. To remove the already bound primary and secondary antibodies, tissue sections were placed in a repair box filled with antigen retrieval solution for microwave heating treatment (heat to above 95 °C and maintain for 15 min). Then, the process was repeated with the next primary antibody, and so on. Sections were observed under an optical microscope. To further determine the expression levels of relative proteins in the tissues, semi-quantitative analysis was conducted by calculating the MOD using Image Pro Plus v. 6.0 software. The calculation method was consistent with that described previously.

The following fluorescence TSA working fluids were used: IF488 (green), IF546 (red), IF440 (cyan), IF594 (orange), and IF647 (yellow), all purchased from Servicebio, Wuhan, Hubei, China. The following antibodies were used: AR, ER, PR, GPX4, HRP-conjugated peroxidase secondary antibodywere purchased from Proteintech, Wuhan, Hubei, China. PRL, IGFBP1, and HOXA10were purchased from Affinity Bio, Changzhou, Jiangsu, China. DPP4 (#YT5707) was purchased from ImmunoWay, Suzhou, Jiangsu, China. The primary antibodies were all diluted at a ratio of 1:100, and the secondary antibody was diluted at a ratio of 1:200. The primary antibodies were all rabbit host polyclonal antibodies, while the secondary antibody was the goat anti-rabbit HRP-conjugated antibody.

### Transmission electron microscopy (TEM)

Cell and tissue samples were fixed overnight in 10% glutaraldehyde and then treated with 1% osmium tetroxide. The samples were dehydrated using a graded series of ethanol and acetone. Ultrathin sections were prepared using an ultramicrotome, embedded in ethoxylate resin, and double-stained with uranyl acetate and lead citrate. The prepared sections were then examined using a TEM (HITACHI, Tokyo, Japan).

### Mitochondrial membrane potential (MMP) assay

Mitochondrial membrane potential was assayed using JC-1 according to the manufacturer’s instructions (Yeasen, Shanghai, China) [47]. Briefly, cells in a six-well cell culture plate were incubated with JC-1 dye at 37 °C under humidified air containing 5% CO_2_ for 15 min in the dark. Then, the cells were washed with a staining buffer. Finally, cells were observed under a microscope. The abnormal in MMP is shown by the emergence of green fluorescence. The presence of red fluorescence suggests normal MMP and cellular health.

### C11-BODIPY^581/591^ examination

Cells were loaded with C11-BODIPY^581/591^ (2 µM) in serum-free medium and incubated at 37 °C for 30 min. After washing, live cells were immediately subjected to imaging. In control cells, strong red fluorescence (590 nm) was observed, indicating minimal lipid peroxidation. In contrast, under ferroptosis-inducing conditions, a pronounced shift toward green fluorescence (510 nm) occurred due to oxidation of the probe, accompanied by a reduction in red signal. This increase in the green-to-red fluorescence ratio quantitatively reflected elevated lipid peroxidation, a hallmark of ferroptosis.

### Correlation analysis between ELISA detection and the ROC curve

To quantitatively analyze the expression levels of DPP4 (#ELK1595, ELK Biotechnology, China), ASCL4 (#ELK9045, ELK Biotechnology, China), TFR1 (#ELK11113, ELK Biotechnology, China), and GPX4 (#ELK4775, ELK Biotechnology, China) in endometrial tissues, we employed the enzyme-linked immunosorbent assay (ELISA). Briefly, endometrial tissue samples were homogenized, and total protein was extracted and quantified using the bicinchoninic acid (BCA) assay. Subsequently, the concentrations of each target protein were measured using commercial specific ELISA kits according to the manufacturer’s protocols. Finally, the obtained protein expression data were subjected to receiver operating characteristic (ROC) curve analysis to evaluate their association with patients’ reproductive outcomes.

### Data and statistical analysis

Statistical analyses were conducted using SPSS (version 24.0, SPSS Inc., Chicago, USA). The normality of the data was assessed using the Shapiro–Wilk test. For data that followed a normal distribution, differences between groups were analyzed by one-way ANOVA and two-way ANOVA followed by Tukey’s post hoc test. Three independent biological samples were used per group. Each assay included three technical replicates within a single experiment, and the entire experiment was independently repeated three times. Results are presented as means ± SEM (standard error of the mean). A *p*-value < 0.05 was considered statistically significant for all comparisons. Mean Difference Value (MD) is statistical value of Tukey’s post hoc test Histograms and statistical distributions were generated in GraphPad Prism software (version 10.1.2, GraphPad software, San Diego, CA, USA).

## Results

### Increased occurrence of ferroptosis in the endometrium of PCOS rats

The PCOS-like rats established by DHEA treatment were used to evaluate the influence of ferroptosis on the endometrial receptivity of PCOS. The successful establishment of the PCOS rat model was evaluated based on the estrous cycle, blood sex hormone levels, and ovarian shape, as described in prior work [19, 48] (Supplementary Fig. 1A-F). We assessed the hormone receptor in the endometrium of PCOS rats.

**Fig. 2:**
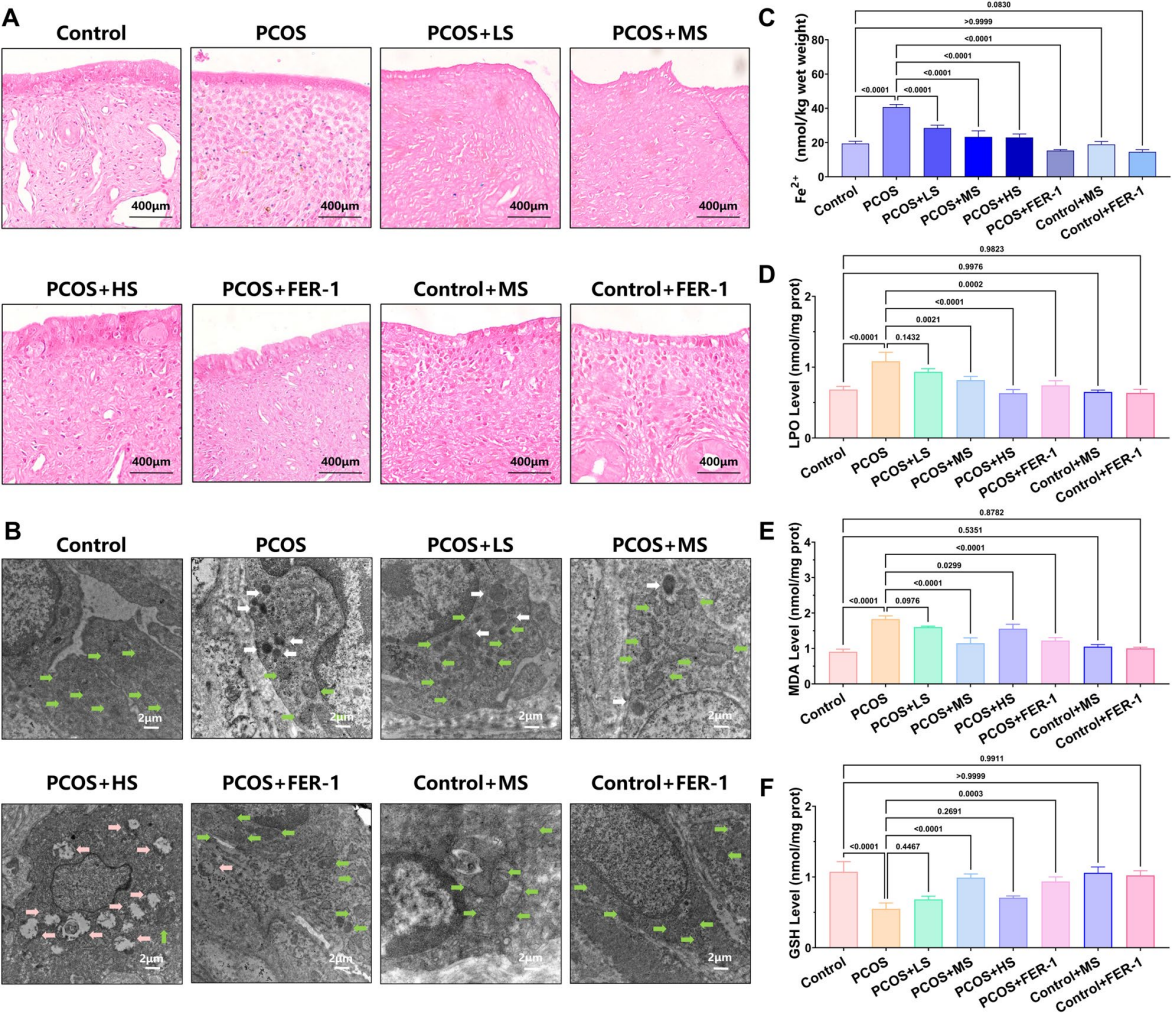
A. Different doses of sitagliptin ameliorated PCOS phenotype and ferroptosis in the endometrium of PCOS rats. A. A. Lillie’s staining of endometrium in each group (the presence of blue particles represented Fe2+ accumulation). B. B. TEM of mitochondria of endometrium each group (magnification, × 8 000. The green arrow represents normal mitochondria, the white arrow represents abnormal mitochondria, and the pink arrow represents abnormal mitochondria undergoing autophagy). C. C. Fe2+ level of endometrium in each group (n = 6), two-way ANOVA test. D. D. LPO level of endometrium in each group (n = 6), two-way ANOVA test. E. E. MDA level of endometrium in each group (n = 6), two-way ANOVA test. F.F. GSH level of endometrium in each group (n = 6), two-way ANOVA test. Data are presented as mean ± SEM. Scale bar: 400 μm in A, 2 μm in B

**Fig. 3:**
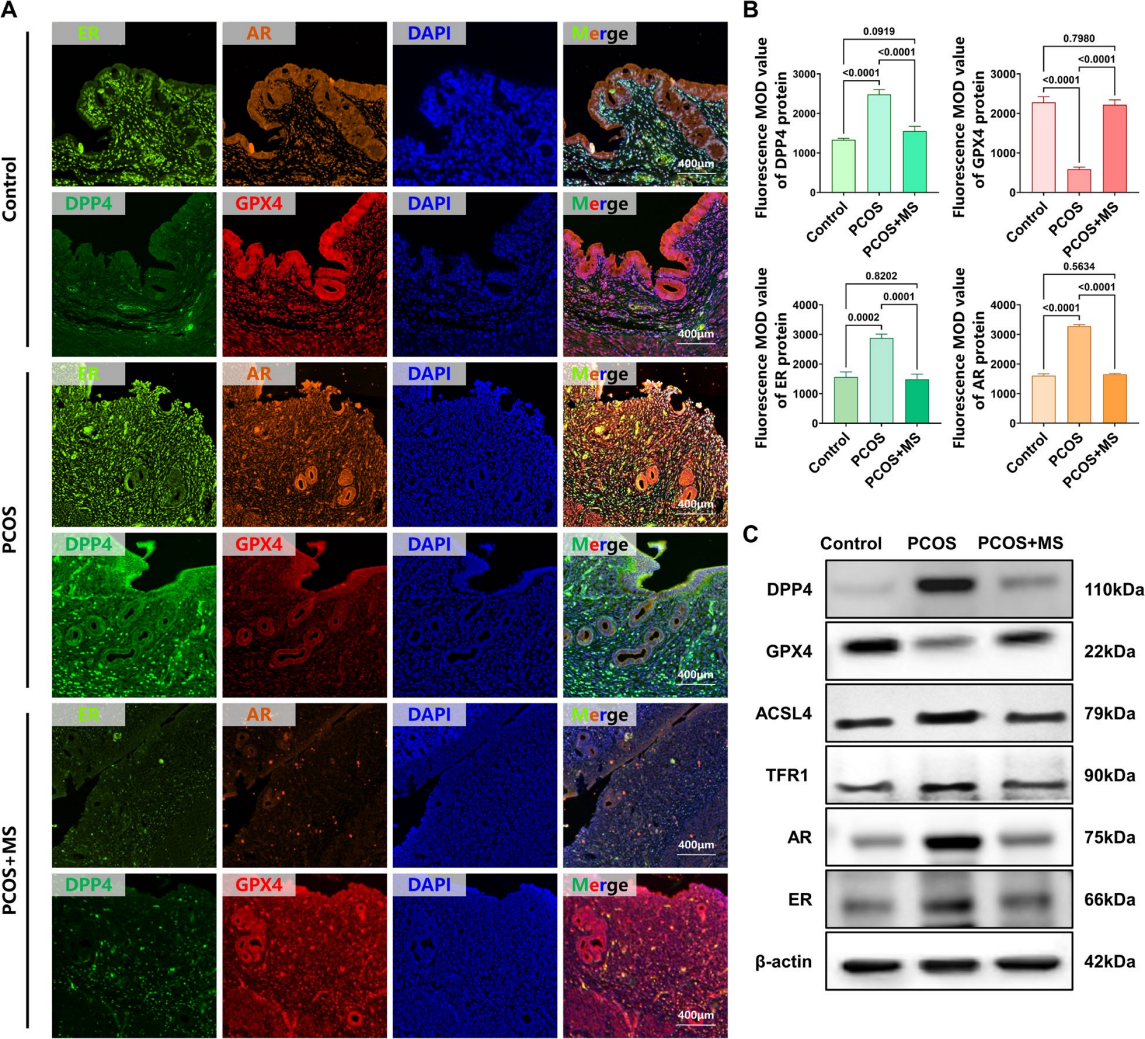
Medium dose of sitagliptin ameliorated PCOS-like phenotype and ferroptosis in the endometrium of PCOS rats. **A ** and** B.** The DPP4, GPX4, ER, and AR each group were determined by immunofluorescence and fluorescence MOD value analysis (n = 6), one-way ANOVA test. **C.** The DPP4, GPX4, ACSL4, TFR1, ER, and AR each group were determined by western blotting. Data are presented as mean ± SEM. Scale bar: 400 μm in A and G

Next, we also evaluated the ferroptosis phenotypes in the endometrium of PCOS rats. The Fe^2+^ content in the endometrium of PCOS rats was increased compared to the Control groups, and the Lillie’s staining showed blue granules, indicating Fe^2+^ accumulation (Fig. 2A and C, MD = − 21.27,* p* < 0.0001,). Importantly, we also observed the abnormal mitochondria in the stromal cells of the endometrium in PCOS rats by using TEM (Fig. 2B). These mitochondria appeared to be smaller in size, with increased density and cristae contraction, which are characteristic features of mitochondria undergoing ferroptosis. Concurrently, LPO (Fig. 2D, MD = − 0.400, *p* < 0.0001) and MDA (MD = − 0.9233, *p* < 0.0001, Fig. 2E) levels were dramatically elevated and GSH (MD = 0.5233, *p* < 0.0001, Fig. 2F) levels were significantly reduced in the endometrium of PCOS rats, suggesting the occurrence of oxidative stress. Moreover, the abundance of the ferroptosis-related protein DPP4,ACSL4 and TFR1 was greatly raised, while the abundance of GPX4 was significantly decreased in the endometrium of PCOS rats compared to the Control groups (Fig. 3A and B). All these findings indicate that ferroptosis occurs in the endometrium of PCOS rats compared to the Control group. In addition, compared to the Control group, the expression levels of ER and AR in the endometrium of PCOS rats were significantly elevated, indicating that the PCOS phenotype could affect hormone expression in the endometrium (Fig. 3 and Supplementary Fig. 3, ER, MD = − 1316, *p* = 0.0002, AR, MD = − 1672, *p* < 0.0001; ER, MD = − 0.6838, *p* < 0.0001, AR, MD = − 0.9317, *p* < 0.0001).

### Sitagliptin alleviated ferroptosis and PCOS phenotypes in the endometrium of PCOS rats

Additionally, we observed that sitagliptin significantly reduced the Fe^2^⁺ content in the endometrium compared to that of the PCOS group (Fig. 2A and C, MD of LS, MS, and HS = 12.23, 17.41, 17.74, all *p* < 0.0001). TEM of endometrial tissue revealed a small number ofin ferroptosis-related mitochondria in the PCOS + LS and MS group. In the PCOS + HS group, mitochondrial autophagy was observed (Fig. 2B). The PCOS + Fer1 group also presented a restoration of normal mitochondrial morphology in the endometrium (Fig. 2B). Notably, the recovery varied among the groups treated with different concentrations of sitagliptin. These results suggest that sitagliptin can inhibit the occurrence of ferroptosis of mitochondria in the endometrium of PCOS rats, although excessive doses may induce mitochondrial autophagy. Following sitagliptin treatment, LPO and MDA levels in the endometrium of PCOS rats were notably decreased, whereas GSH levels were significantly increased (Fig. 2D, MD of LS, MS, and HS = 0.1500, 0.2667, 0.4500, *p* = 0.1432, *p* = 0.0021, *p* < 0.0001, Fig. 2E, MD of LS, MS, and HS = 0.2267, 0.6833, 0.2733, *p* = 0.0976, *p* < 0.0001,* p* = 0.0299, Fig. 2F, MD of LS, MS, and HS = − 0.1333, −0.4400, − 0.1567, *p* = 0.4467, *p* < 0.0001,* p* = 0.2691). Compared with the PCOS + LS group, the PCOS + MS group demonstrated a more rapid improvement in the reduction of divalent iron content, antioxidant imbalance, and mitochondrial damage. Additionally, oxidative stress and mitochondrial autophagy associated with high drug concentrations were more effectively mitigated in the PCOS + MS group than in the PCOS + HS group. Compared with the PCOS group, sitagliptin improved the disruption of the estrous cycle in PCOS-like rats (Supplementary Fig. 4). Based on these results, we selected 10 mg/kg sitagliptin in the PCOS + MS group as the appropriate dosage for further research.

Sitagliptin treatment effectively reduced the expression levels of ER and AR in the endometrium of PCOS rats. Additionally, sitagliptin reversed the decrease in GPX4 levels and reduced the rise of DPP4. The results from immunofluorescence and western blotting revealed the same trend (Fig. 3A and B, DPP4, MD = 928.0, *p* < 0.0001, GPX4, MD = − 1629, *p* < 0.0001, ER, MD = 1397, *p* = 0.0001, AR, MD = 1626, *p* < 0.0001; Fig. 3C and Supplementary Fig. 3, DPP4, MD = 0.7159, *p* < 0.0001, ACSL4, MD = 0.5235, *p* = 0.0103, TFR1, MD = 0.6070, *p* = 0.0003, GPX4, MD = − 0.7319, *p* < 0.0001, ER, MD = 0.6216, *p* = 0.0002, AR, MD = 0.7506, *p* < 0.0001).

### Sitagliptin treatment ameliorated on the endometrial receptivity in PCOS rats

**Fig. 4:**
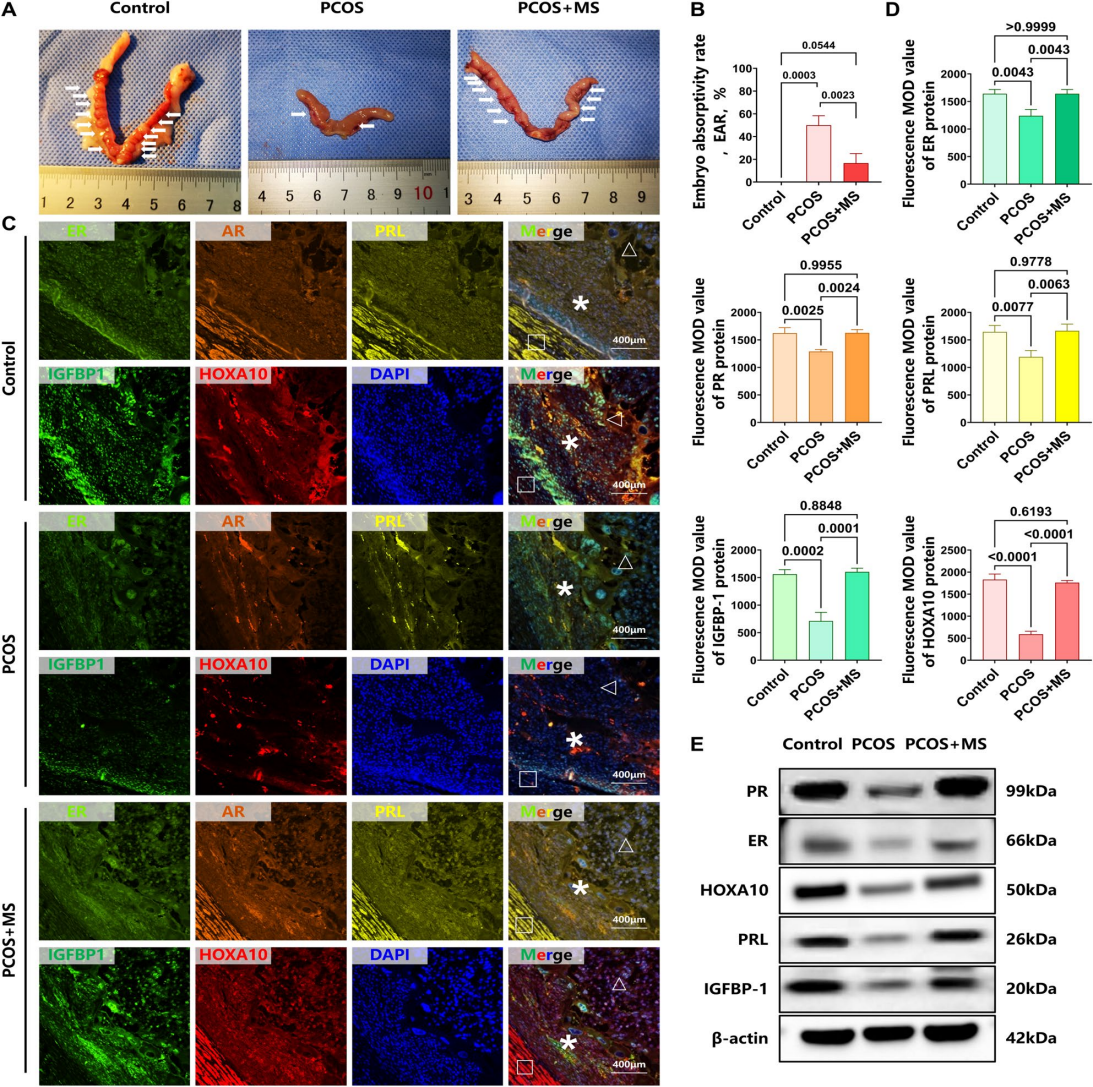
Sitagliptin treatment ameliorated the endometrial receptivity in PCOS rats. **A**. The rat pregnancy model, with the uterus at GD 10 of pregnancy respectively in Control, PCOS, and PCOS + MS groups. **B.** Embryo absorptivity rate in each group (n = 6). **C **and** D.** The ER, PR, PRL, IGFBP-1, and HOXA10 in the pregnancy endometrium in each group were determined by immunofluorescence (n = 6), one-way ANOVA test. * represented decidua basalis. $\triangle$ represented placenta. $\square$ represented myometrium. **E.** The ER, PR, PRL, IGFBP-1, and HOXA10 in each group were determined by western blotting. Data are presented as mean ± SEM. Scale bar: 400 μm in E

In this study, we validated the uteri of rats on the GD 10, focusing on the number of intrauterine embryos. Compared to the Control group, the PCOS group exhibited significantly smaller embryo volumes due to ischemia, hemorrhage, and necrosis, resulting in a marked difference from normal embryos. Additionally, the absorbed embryos appeared dark brown, while the normal embryos were pink. The results from the PCOS + MS group showed embryo numbers and morphology similar to the Control group, indicating that sitagliptin significantly improves the number and morphology of embryos in the PCOS group. The results indicated that the EAR in the PCOS group was significantly higher than that in the Control group (Fig. 4A and B, MD = − 0.5, *p* = 0.0003). In contrast, the PCOS + MS group exhibited a significantly lower EAR compared to the PCOS group (Fig. 4A and B, MD = 0.3333, *p* = 0.0023).

Compared to the Control group, the PCOS group exhibited decreased levels of ER, PR, and PRL, indicating incomplete decidualization(Fig. 4C and D, MD of ER, PR, and PRL = 400.1, 334.2, 454.9, *p* = 0.0043,* p* = 0.0025, *p* = 0.0077; Fig. 4Eand Supplementary Fig. 5,MD of ER, PR, and PRL = 0.6140, 0.4345, 0.4203, *p* < 0.0001, *p* = 0.0032, *p* = 0.0009). In contrast, the PCOS + MS group showed significant increases in the endometrial decidualization markers ER, PR, and PRL when compared to the PCOS group (Fig. 4C and D, MD of ER, PR, and PRL = − 400.1, − 339.3, − 474.3, *p* = 0.0043, *p* = 0.0024, *p* = 0.0063; Fig. 4E and Supplementary Fig. 5, MD of ER, PR, and PRL = − 0.5009, − 0.4169, − 0.4156, *p* < 0.0001, *p* = 0.0039, *p* = 0.0009;). Regarding endometrial receptivity indicators, levels of IGFBP-1 and HOXA10 were significantly lower in the PCOS group compared to the Control group, suggesting abnormal endometrial receptivity following decidualization (Fig. 3C and D, MD of IGFBP-1 and HOXA10 = 0.8386, 0.6601, *p* < 0.0001, *p* < 0.0001; Fig. 3E and F, MD of IGFBP-1 and HOXA10 = 849.2, 1240, *p* = 0.0002, *p* < 0.0001). Following sitagliptin intervention, the expression levels of IGFBP-1 and HOXA10 in the PCOS + MS group improved relative to the PCOS group (Fig. 4C and D, MD of IGFBP-1 and HOXA10 = − 891.5, − 1172, *p* = 0.0001, *p* < 0.0001; Fig. 4E and Supplementary Fig. 5, MD of IGFBP-1 and HOXA10 = − 0.8144, − 0.6055, *p* < 0.0001, *p* < 0.0001).

### Elevated ferroptosis in the HD DHEA-induced T-HESCs

**Fig. 5:**
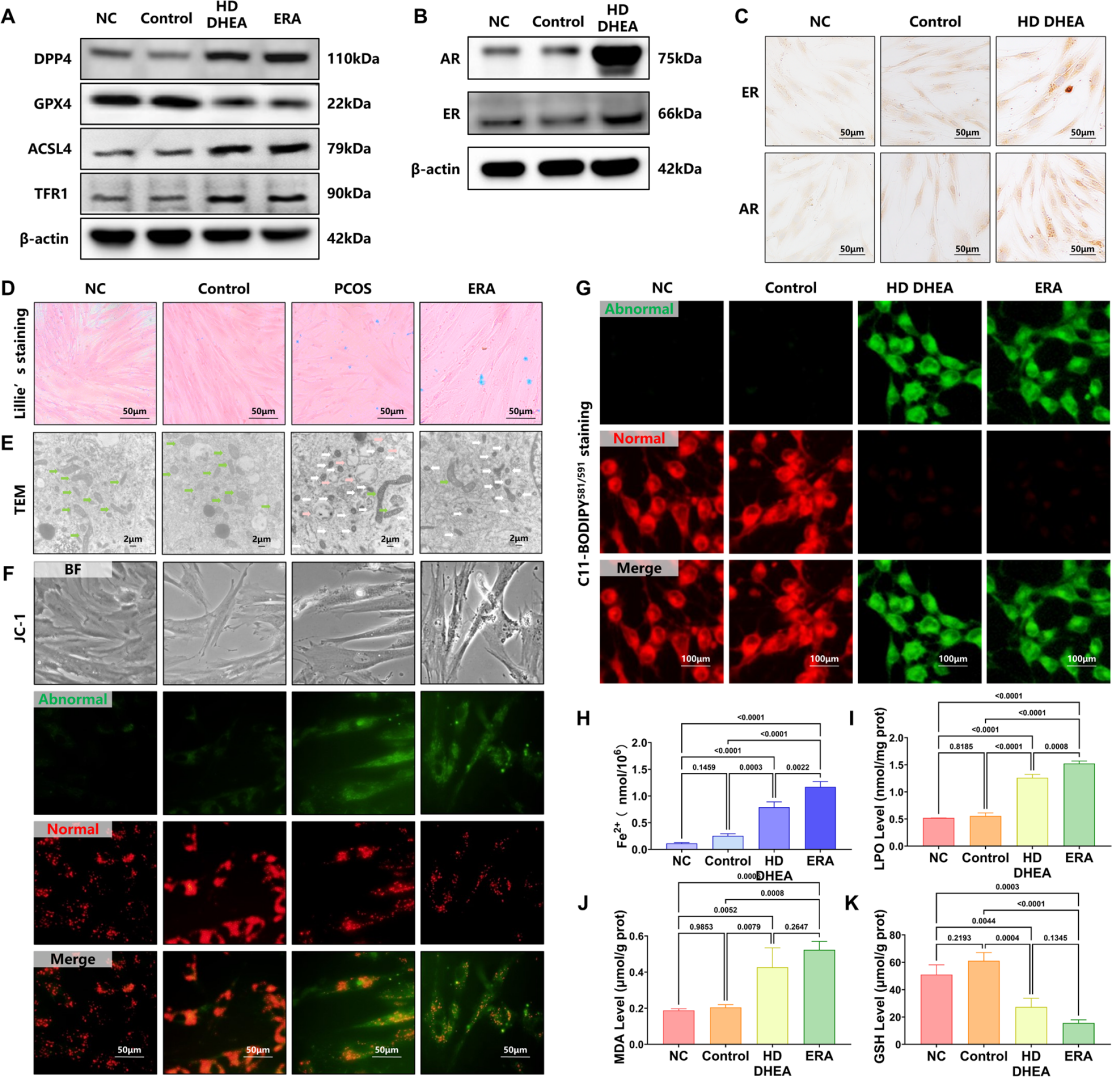
Elevated ferroptosis in the HD DHEA T-HESCs **A.** The DPP4, ACSL4, TFR1, and GPX4 in each group were determined by western blotting. **B.** The ER and AR in each group were determined by western blotting. **C.** The ER and AR in each group were determined by immunohistochemistry. **D.** Lillie’s staining of endometrium in NC, Control, PCOS, and ERA group. **E.** TEM of mitochondria of endometrium in NC, Control, PCOS, and ERA group (magnification, × 10 000. The green arrow represents normal mitochondria, the white arrow represents abnormal mitochondria, and the pink arrow represents abnormal mitochondria undergoing autophagy). **F.** The mitochondrial membrane potential was determined by JC-1 in each group. (The green fluorescence suggests a drop in mitochondrial membrane potential. The red fluorescence suggests normal mitochondrial membrane potential and cellular health.)** G.** C11-BODIPY^581/591^ staining (The green fluorescence suggests the LPO increased. The red fluorescence suggests the LPO decreased). **H.** Fe^2+^ level in each group, one-way ANOVA test. **I.** LPO level in each group, one-way ANOVA test. **J.** MDA level in each group, one-way ANOVA test. **K.** GSH level in each group, one-way ANOVA test. Data are presented as mean ± SEM. Scale bar: 50 μm in C and F, 100 μm in G,2 μm in E

In our study, we first established and validated the status of HD DHEA-induced T-HESCs. To ascertain the occurrence of ferroptosis in HD DHEA-induced T-HESCs, using erastin as a positive control group. We examined our screened ferroptosis proteins (GPX4, ACSL4, TFR1, and DPP4) by western blotting, which showed that GPX4 was reduced, and ACSL4, TFR1, DPP4 was increased significantly in the HD DHEA and ERA groups compared with Control group (Fig. 5A and Supplementary Fig. 6 A, *p* < 0.0001). The findings indicated that ferroptosis might have occurred in DHEA-induced HD DHEA-induced T-HESCs. Sex hormone receptor-related protein expression was measured using immunohistochemistry and western blotting. There is no significant difference in the expression levels of ER and AR between the NC and Control group (Fig. 5B and Supplementary Fig. 6B, MD of ER and AR = were significantly lower in the PCOS group compared to the Control group, suggesting abnormal endometrial 7.140, were significantly lower in the PCOS group compared to the Control group, suggesting abnormal endometrial 6.732, *p* = 0.0592, *p* = 0.0863; Fig. 5C and Supplementary Fig. 6 C, MD of AR and ER = were significantly lower in the PCOS group compared to the Control group, suggesting abnormal endometrial 0.02647, − 0.6486, *p* = 0.9216, *p* = 0.0937). ER and AR exhibited considerably higher expression levels in HD DHEA-induced T-HESCs compared to the Control group (Fig. 5B and Supplementary Fig. 6B, MD of ER and AR = were significantly lower in the PCOS group compared to the Control group, suggesting abnormal endometrial 21.41, were significantly lower in the PCOS group compared to the Control group, suggesting abnormal endometrial 40.10, *p* < 0.0001, *p* < 0.0001; Fig. 5C and Supplementary Fig. 6 C, MD of AR and ER = − 1.014, were significantly lower in the PCOS group compared to the Control group, suggesting abnormal endometrial 0.6486, *p* < 0.0001, *p* < 0.0001).

The Lillie’s staining showed blue-stained granules in HD DHEA and ERA groups, suggesting substantial Fe^2^⁺ accumulation in T-HESCs from the HD DHEA group (Fig. 5D). The cellular Fe^2^⁺ concentration was measured using colorimetric methods in each group. Fe^2^⁺ levels were marginally higher in the Control group compared to the NC group, but the difference was not significant (Fig. 5H, MD = were significantly lower in the PCOS group compared to the Control group, suggesting abnormal endometrial 0.1398, *p* = 0.2033). The HD DHEA and ERA groups had greater Fe^2^⁺ levels than the NC and Control groups (Fig. 5H = 121.33, *p* < 0.0001), with the HD DHEA group having lower levels than the ERA group (Fig. 4E, MD = were significantly lower in the PCOS group compared to the Control group, suggesting abnormal endometrial 0.3285, *p* = 0.0038). TEM showed that mitochondria of HD DHEA-induced T-HESCs had decreased volume, shortened cristae, and increased membrane density, indicating ferroptosis in the HD DHEA group (Fig. 5E). Both HD DHEA and ERA groups had more green fluorescent mitochondria and less red luminous mitochondria in MMP assay by using JC-1 tests, suggesting more damaged mitochondria. The ERA group had comparable mitochondrial damage (Fig. 5F), demonstrating HD DHEA-induced T-HESC mitochondrial impairment intensity. Higher LPO (Fig. 5G and I, F = 318.40 *p*<0.0001) and MDA (Fig. 5I, F = 22.33, *p* = 0.0003) levels in HD DHEA and ERA group than in NC and Control groups. The HD DHEA and ERA groups showed a substantial drop in GSH levels compared to the NC and Control groups (Fig. 5J, F = 39.66, *p*< 0.0001). LPO (MD = − 0.03499, *p* = 0.8185), MDA (MD = − 0.01658, *p* = 0.9853), and GSH (MD = were significantly lower in the PCOS group compared to the Control group, suggesting abnormal endometrial 10.07, *p* = 0.2193) levels were similar in the NC and Control groups (Fig. 5I-5). In summary, there is an occurrence of ferroptosis in the HD DHEA-induced T-HESCs induced by our methods.

### Regulating DPP4 may improve the PCOS phenotype by influencing ferroptosis

**Fig. 6:**
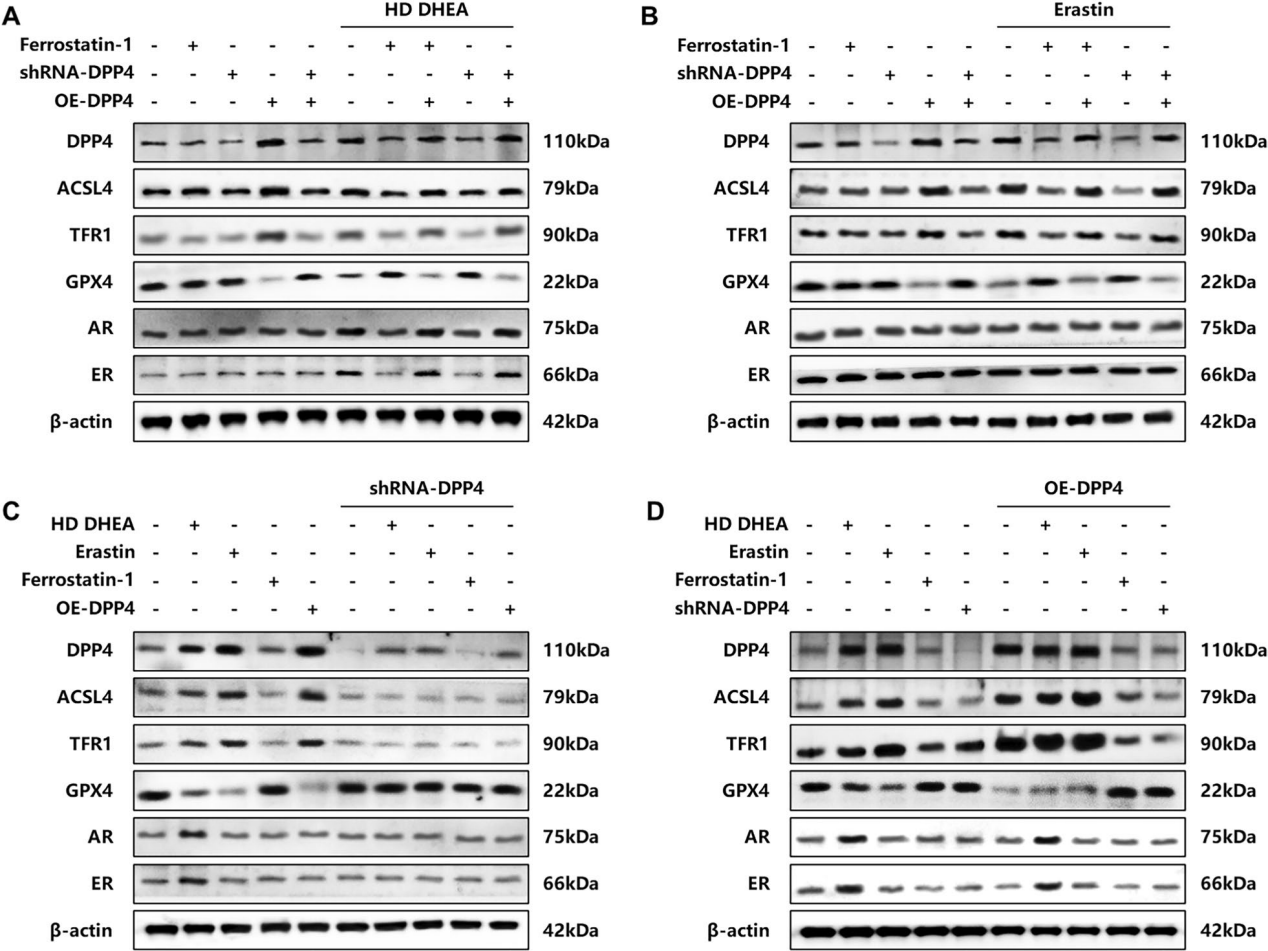
Downregulating DPP4 ameliorated PCOS-like status and the occurrence of ferroptosis. **A.** DPP4 knockdown and ferrostatin-1 rescue HD DHEA-induced PCOS phenotype and ferroptosis in T-HESCs. **B.** DPP4 knockdown and ferrostatin-1 rescue erastin-induced ferroptosis in T-HESCs. **C.** HD DHEA, erastin, and OE-DPP4 rescue DPP4 knockdown, inhibiting PCOS phenotype and ferroptosis in T-HESCs. **D.** HD DHEA, erastin, and OE-DPP4 aggravate DPP4 overexpression-induced PCOS phenotype and ferroptosis in T-HESCs

To elucidate the functional role of DPP4 in HD DHEA-induced ferroptosis and hormonal receptor dysregulation, a series of rescue experiments were conducted. It was observed that in HD DHEA-induced model cells, AR and ER expression was significantly up-regulated. These changes were accompanied by characteristic features of ferroptosis, including depletion of GPX4 and elevation of ASCL4 and TFR1 (Fig. 6A). However, upon treatment with either DPP4 knockdown (shRNA-DPP4) or the ferroptosis inhibitor ferrostatin-1, not only was the ferroptotic phenotype effectively reversed, but the aberrant AR and ER expression levels were also unexpectedly normalized. Conversely, when DPP4 was overexpressed (OE-DPP4) in the presence of DHEA induction, the suppressed ferroptosis indicators and abnormal hormone receptor expression caused by knockdown or inhibition were restored (Fig. 6A, 6B and 6 C). Further investigation revealed that both DPP4 knockdown and ferrostatin-1 treatment significantly alleviated the aggravated ferroptosis induced by either the ferroptosis inducer erastin or OE-DPP4. It is noteworthy that although erastin treatment alone successfully induced ferroptosis, it did not cause significant alterations in AR or ER expression, indicating that ferroptosis per se is not the direct cause driving changes in hormonal receptor expression (Fig. 6C and 6D).

**Fig. 7:**
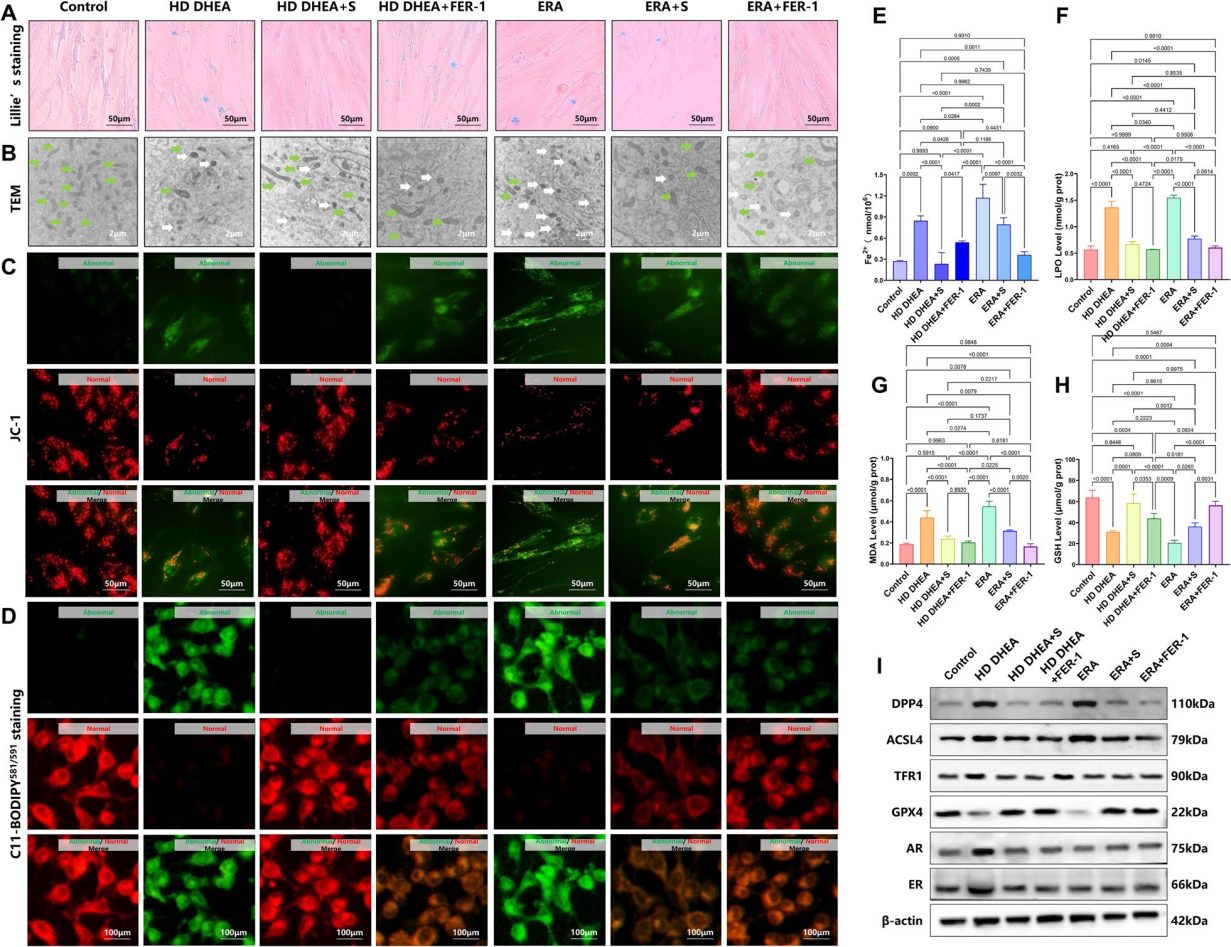
Sitagliptin ameliorated the occurrence of ferroptosis. **A.** Lillie’s staining of the endometrium in each group. **B.** TEM of mitochondria of endometrium in each group (magnification, × 10 000. The green arrow represents normal mitochondria; the white arrow represents abnormal mitochondria). **C.** The mitochondrial membrane potential of JC-1 in each group. **D.** C11-BODIPY^581/591^ staining (The green fluorescence suggests the LPO increased. The red fluorescence suggests the LPO decreased). **E.** Fe^2+^ level in each group (n = 3), two-way ANOVA test. **F.** LPO level in each group (n = 3), two-way ANOVA test. **G.** MDA level in each group (n = 3), two-way ANOVA test. **H.** GSH level in each group (n = 3), two-way ANOVA test. **I.** The expression levels of DPP4, ACSL4, TFR1, GPX4, ER, and AR in each group were determined by western blotting and analyzed using a two-way ANOVA test (n = 3). Data are presented as mean ± SEM. Scale bar: 2 μm in B, 50 μm in C, 100 μm in D

Compared with Control group, Fe^2^⁺ content measurements and Lillie staining revealed significant iron accumulation in the HD DHEA and ERA groups (Fig. 7A and 7E, MD of HD DHEA and ERA = − 0.5757, − 0.9017, *p* = 0.0002, *p* < 0.0001). However, adding sitagliptin or FER-1 to the PCOS group significantly reduced Fe^2^⁺ levels (Fig. 7A and E, MD of HD DHEA + S and HD DHEA + FER-1 = 0.6140, 0.3065, *p* < 0.0001, *p* = 0.0426). Similarly, compared with ERA group, Fe^2^⁺ levels also decreased significantly in the ERA + S and ERA + FER-1 groups (Fig. 7A and E, MD of ERA + S and ERA + FER-1 = 0.3775, 0.8093, *p* = 0.0097, *p* < 0.0001). These results indicate that sitagliptin effectively alleviates iron accumulation in HD DHEA-induced cells and suppresses ferroptosis.

TEM further confirmed that it was difficult to observe ferroptosis mitochondria in the HD DHEA + S and HD DHEA + FER-1 groups (Fig. 7B). The MMP assays also demonstrated an increase in mitochondria with normal membrane potential in above groups by using JC-1 (Fig. 7C). Compared with HD DHEA and ERA group, LPO, MDA, and GSH assays showed that sitagliptin reduced lipid peroxidation products (Fig. 7D and 7 F, MD of HD DHEA + S and ERA + S = 0.6935, 0.7744, *p* < 0.0001, *p* < 0.0001; Fig. 7G, MD of HD DHEA + S and ERA + S = 0.2019, 0.2322, *p* < 0.0001, *p* < 0.0001) and increased antioxidant levels (Fig. 7H, MD of PCOS + S and ERA + S = − 27.68, − 15.49, *p* = 0.0001, *p* = 0.0260), suggesting it mitigates oxidative imbalance and inhibits ferroptosis in HD DHEA-induced cells.

Western blotting analysis showed that, compared to the control group, DPP4, ACSL4 and TFR1 expression (Fig. 7I and Supplementary Fig. 7,* p* < 0.0001) was increased and GPX4 expression (Fig. 7H, MD of PCOS and ERA = 0.5684, 0.7660, *p* < 0.0001,* p* < 0.0001) decreased in both the HD DHEA and ERA groups. Compared with HD DHEA group, sitagliptin reduced DPP4, ACSL4 and TFR1 expression and increased GPX4 levels, while also reducing the expression of ER and AR (Fig. 7I and Supplementary Fig. 7, MD of DPP4, GPX4, ER, and AR = 0.7196, − 0.5368, 0.7215, 0.7455, all* p* < 0.0001). Similarly, compared with HD DHEA group, ferrostatin were significantly lower in the PCOS group compared to the Control group, suggesting abnormal endometrial 1 also lowered ER and AR expression in HD DHEA-induced T-HESCs, suggesting that inhibiting ferroptosis may improve the PCOS phenotype (Fig. 7I and Supplementary Fig. 7, MD of ER, and AR = 0.6588, 0.6357, all* p* < 0.0001). Meanwhile, we also found that compared with the Control group, the expression levels of AR and ER in the ERA group did not increase or decrease (Fig. 7I and Supplementary Fig. 7, MD of ER, and AR = 0.2217, 0.1128,* p* = 0.1345,* p* = 0.0615), suggesting that erastin alone in normal T-HESCs does not alter AR or ER expression, and therefore modulating ferroptosis in the ERA group does not affect hormonal changes.

### Sitagliptin could ameliorate endometrial receptivity in decidualized HD DHEA-induced T-HESCs cells

**Fig. 8:**
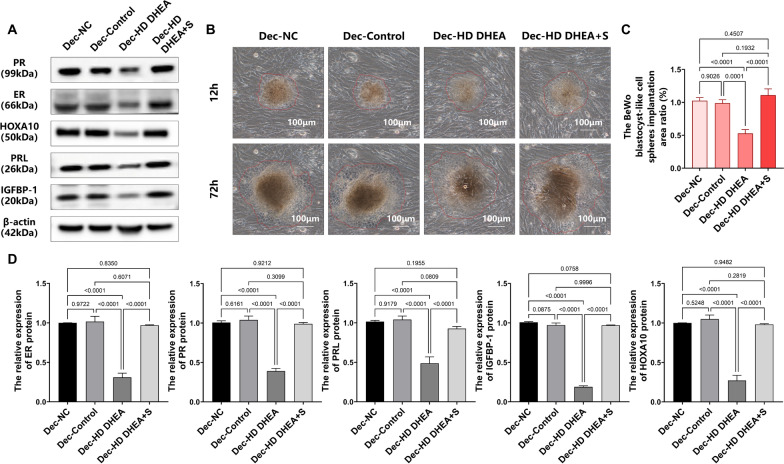
Sitagliptin improved endometrial receptivity in decidualized HD DHEA-induced T-HESCs**. A **and** D.** ER, PR, PRL, IGFBP1, and HOXA10 in each group were determined by western blotting and analysis (n = 3), one-way ANOVA test. **B.** The area of each group of the BeWo blastocyst-like cell spheres was measured in vitro at 12 and 72 h. **C.** The ratio of the invasion area of the BeWo blastocyst-like cell spheres in each group (n = 3), one-way ANOVA test. Data are presented as mean ± SEM. Scale bar: 100 μm in B

The results indicated that the expression of decidualization-related proteins (ER, PR, and PRL) and endometrial receptivity markers (IGFBP were significantly lower in the PCOS group compared to the Control group, suggesting abnormal endometrial 1 and HOXA10) in the Dec-HD DHEA group was significantly lower compared to the Dec-Control groups (Fig. 8A and D, MD of ER, PR, PRL, IGFBP-1, and HOXA10 = 0.7063, 0.6490, 0.5518, 0.7828, 0.7776, all* p* < 0.0001). This suggests that the HD DHEA-induced model cells faced challenges in decidualization induction. In contrast, the Dec-HD DHEA + S group exhibited significant increases in ER, PR, PRL, IGFBP-1, and HOXA10 expression(Fig. 8A and D, MD of ER, PR, PRL, IGFBP-1, and HOXA10 = −0.6614, − 0.5984, − 0.4382, − 0.7815, − 0.7112, all* p* < 0.0001), showing no significant differences when compared to the Dec-Control groups(Fig. 8A and D, MD of ER, PR, PRL, IGFBP-1, and HOXA10 = 0.04485, 0.05058, 0.1135, 0.001299, 0.06644,* p* = 0.6071, *p* = 0.3099,* p* = 0.0809,* p* = 0.9996,* p* = 0.2819), indicating that sitagliptin effectively induces decidualization and improves endometrial receptivity.

The results demonstrated that the BeWo blastocyst-like cell sphere invasion area in the Dec-HD DHEA group was significantly smaller than that in the Dec-Control group after 72 h of implantation (Fig. 8B and C, MD = 0.4606,* p* = 0.0001). Conversely, the Dec-HD DHEA + S group showed a significantly larger invasion area compared to the Dec-HD DHEA group (Fig. 8B and C, MD = −0.5807,* p* < 0.0001). These findings suggest that sitagliptin can enhance endometrial receptivity in HD DHEA-induced model T-HESCs and promote the invasion ability of the BeWo blastocyst-like cell spheres.

### Sitagliptin could improve reproductive outcomes in PCOS patients

**Fig. 9:**
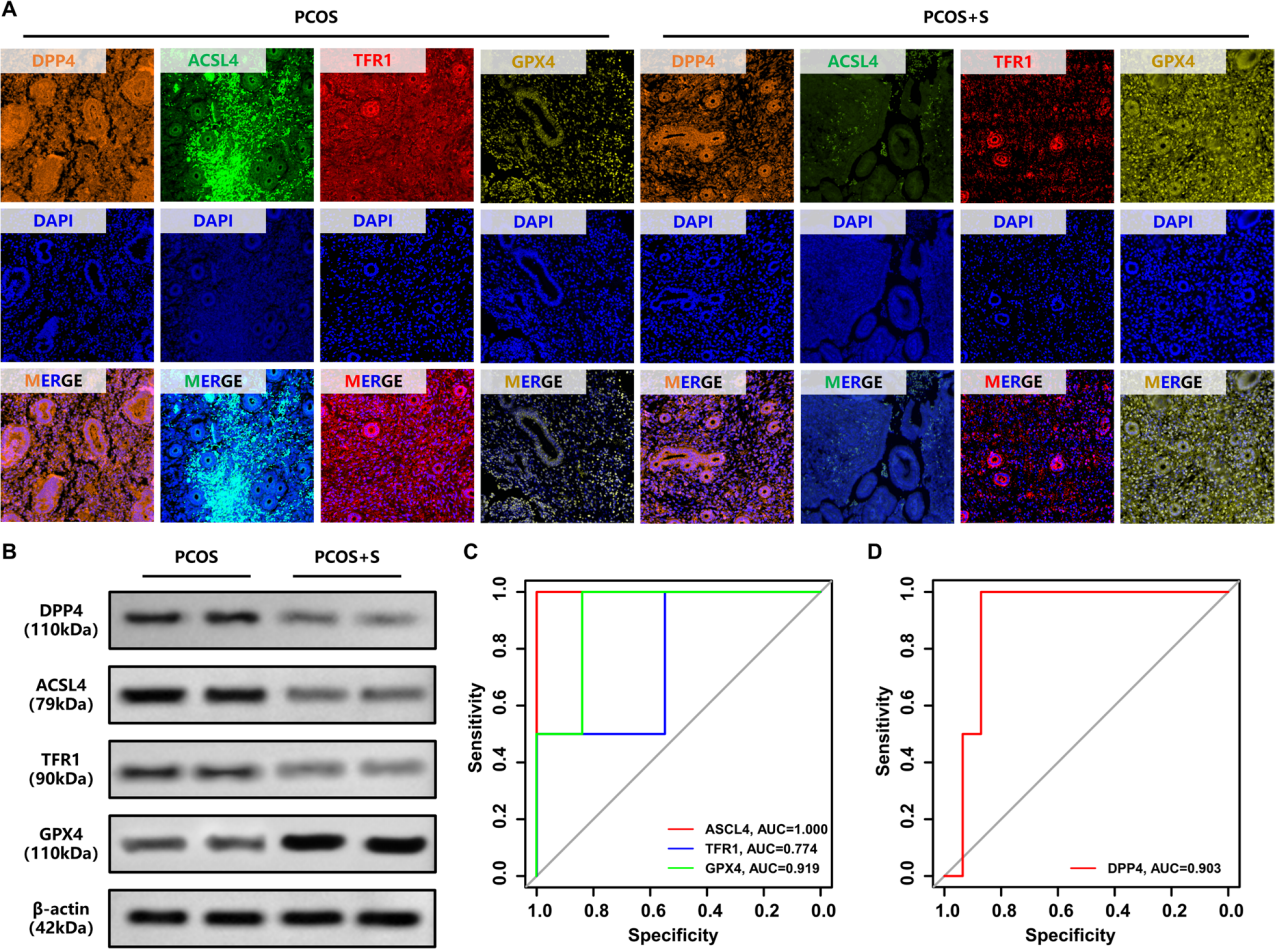
Sitagliptin improved reproductive outcomes in PCOS patients. **A.** The DPP4, ACSL4, TFR1, and GPX4 in each group were determined by immunofluorescence. **B.** The DPP4, ACSL4, TFR1, and GPX4 in each group were determined by western blotting. **C.** Correlation of ASCL4, TFR1, and GPX4 expression with reproductive outcomes by ROC curve analysis in PCOS patients (n = 33). **D.** Correlation of DPP4 expression with reproductive outcomes by ROC curve analysis in PCOS patients (n = 33)

Through the analysis of the clinical data and endometrial samples we collected, this study focused on the therapeutic effects of sitagliptin on endometrial conditions and reproductive outcomes in PCOS patients. Compared with pre-treatment measurements, PCOS patients treated with sitagliptin showed a significant reduction in DPP4 activity in endometrial tissue (Fig. 9A, B, and Supplementary Fig. 8, t value of DPP4 = 6.020,* p* = 0.0038). We also observed a marked increase in GPX4 expression, accompanied by substantial decreases in ACSL4 and TFR1, indicating a favorable modulation of key ferroptosis markers (Fig. 9A, B, and Supplementary Fig. 8, t value of ASCL4, TFR1, and GPX4 = 5.064, 7.956, 9.741,* p* = 0.0072, *p* = 0.0014, *p* = 0.0006). Clinical reproductive outcomes confirmed the potential of the sitagliptin therapy, as among the 33 patients included, 31 achieved live births, and the clinical pregnancy rate and live birth rate in the treatment group (PCOS + S) were significantly higher than before sitagliptin treatment (Fig. 9A and B, AUC of ROC in ASCL4, TFR1, GPX4, and DPP4 = 1.000, 0.774, 0.919, 0.903). The findings suggested that sitagliptin effectively inhibits ferroptosis in the endometrium of PCOS patients, thereby potentially improving endometrial function and receptivity. Correspondingly, recorded reproductive outcomes showed that the clinical pregnancy rate and live birth rate were significantly higher in the PCOS + S group than before sitagliptin treatment. The sitagliptin was suggested to play a beneficial role in improving adverse reproductive outcomes in PCOS patients by modulating endometrial DPP4 activity and suppressing ferroptosis.

## Discussion

Building on our previous research that identified ferroptosis as a key mechanism underlying endometrial abnormalities in PCOS [18], this study aimed to elucidate the role of DPP4-dependent ferroptosis. Endometrial research was challenging due to the tissue’s complex dynamic characteristics, including the multilayer hormonal regulation networks [49–51]. This is particularly true for PCOS, where the lack of a well-established in vitro HD-DHEA-induced stromal cell model and the physiological differences between rodent estrous cycles and the human menstrual cycle present significant hurdles [14, 52]. Despite these limitations, research on the link between PCOS and the endometrium has been conducted [12, 53–56]. Owing to ovulation dysfunction and limited menstruation, PCOS patients have difficulty entering the luteal phase without external treatment intervention. Bearing this in mind, assessing and researching endometrial receptivity defects in PCOS should begin during the proliferative phase and continue until decidualization is induced. Using this model, we confirmed that ferroptosis occurs in the endometrium and is predominantly localized to ESCs. This finding highlights ESCs not only as crucial for receptivity but also as a primary site of ferroptosis in PCOS.

Before exploring the mechanisms through which DPP4 influences endometrial receptivity, we constructed a hyperandrogenism-induced cell model to clarify the association between ferroptosis and endometrial receptivity in PCOS. In recent years, researchers have increasingly concentrated on employing cell models to further the knowledge of the pathogenesis and therapeutic strategies for PCOS [57]. The creation of a PCOS endometrial stromal cell model offers a new viewpoint for PCOS research. Many pertinent studies utilize primary cells from PCOS patients; however, this approach is characterized by variability and inadequate reproducibility, and the lack of hormonal intervention may result in the loss of PCOS-associated hormone receptor phenotypes, consequently constraining the validity of the research [58–60]. Furthermore, a standardized in vitro model for PCOS endometrial stromal cells is currently lacking, with significant methodological inconsistencies hindering progress [14, 52]. To address this, we developed a dehydroepiandrosterone (DHEA)-induced model using the T-HESC cell line. This approach offers several critical advantages: (1) it allows for the precise evaluation of the androgen receptor (AR) signaling pathway in isolation, minimizing the confounding effects of estrogen; (2) DHEA exhibits high metabolic stability in culture, ensuring consistent and controllable dosing; and (3) it provides a highly reproducible platform for mechanistic studies. As this study focuses on the direct effects of hyperandrogenism, the DHEA-induced model is particularly suitable for elucidating the underlying molecular mechanisms in T-HESCs. The DHEA concentration was selected based on clinical relevance. Literature indicates that serum-free testosterone levels in PCOS patients do not exceed twice the normal average [37]. Accordingly, we calibrated our in vitro concentration to reflect this pathophysiological range. The establishment of this well-defined model provides a robust and reproducible foundation for our mechanistic investigation and future research.

In HD DHEA-induced cells, oxidative species (LPO and MDA) and antioxidant consumption (GSH) increase, indicating a persistent state of antioxidant imbalance similar to that induced by erastin. In contrast, erastin induction itself did not alter AR or ER expression, suggesting that ferroptosis does not directly affect hormone receptor expression. Instead, the PCOS phenotype may be the upstream trigger of ferroptosis. Utilizing the established HD DHEA-induced PCOS cell model, we identified DPP4 as a potentially crucial protein influencing ferroptosis and endometrial receptivity in patients with PCOS. The expression of DPP4 was markedly increased in HD DHEA-induced cells, indicating its potential as a target for amplified the PCOS phenotype. DPP4 is a membrane-bound enzyme expressed in numerous cell types that functions to cleave bioactive peptides [61]. Recent investigations had established that DPP4 was integral to ferroptosis, and participated in the regulatory mechanisms of this process [62]. Clinically, DPP4 activity and concentration were significantly increased in the blood of PCOS patients and favorably correlated with increased levels of free testosterone [63, 64]. This suggests that DPP4 may link hyperandrogenism to ferroptosis in PCOS. One of the most significant findings of this study is the identification of DPP4 as playing a central role in regulating both DHEA-induced ferroptosis and hormone receptor expression in endometrial cells. Our rescue experiments clearly demonstrated that inhibition of DPP4 or ferroptosis not only alleviated cellular stress but also concurrently corrected the imbalanced expression of AR and ER. In contrast, overexpression of DPP4 produced the opposite effect. This strongly supports that DPP4 serves as a critical node linking hyperandrogen signaling, ferroptosis, and hormonal receptor dysfunction. A particularly noteworthy observation was that although inhibition of ferroptosis simultaneously ameliorated abnormal hormone receptor expression, direct induction of ferroptosis did not per se upregulate AR or downregulate ER expression. This indicates that ferroptosis is likely an executive event downstream of the DHEA-DPP4 signaling axis, rather than a direct upstream trigger of altered receptor expression. It is speculated that DHEA, potentially through activating DPP4 expression, may coordinately regulate multiple downstream processes in parallel: directly or indirectly influencing AR/ER transcription or stability on one hand, and triggering ferroptosis through promoting lipid peroxidation, on the other. These two processes may crosstalk, for instance, by sharing a common oxidative stress microenvironment, thereby enabling inhibition of either DPP4 or ferroptosis to produce synergistic rescue effects. This mechanistic model highlights the central position of DPP4 in endometrial dysfunction under hyperandrogenic conditions. Targeting DPP4 not only inhibited the destructive process of ferroptosis but also surprisingly restored normal responsiveness to estrogen and androgens. This provides a dual theoretical basis and a highly promising treatment strategy, which use of DPP4 inhibitors such as sitagliptin for managing endometrial receptivity defects associated with PCOS. Nevertheless, the specific molecular intermediates through which DPP4 regulates AR/ER expression remain to be fully elucidated in future studies. Our findings also indicated that elevated DPP4 expression in endometrial stromal cells induces ferroptosis, whereas diminished DPP4 expression mitigates ferroptosis and the impact of androgens in PCOS model cells. Furthermore, since elevated androgen levels in patients with PCOS had been demonstrated to suppress aromatase expression. Inhibition of DPP4 expression could increase aromatase levels in the granulosa cells of PCOS patients, facilitating the conversion and reduction of androgens [65–67]. Variations in DPP4 activity may also impact testosterone production by modulating adipokines and adiponectin [68]. Therefore, downregulating DPP4 expression appears to correct abnormal hormone receptor expression in HD-DHEA-induced model cells by effectively inhibiting ferroptosis. Consequently, our findings underscore the significance of DPP4in the pathophysiology of PCOS and warrants further investigation as a potential therapeutic target.

Building upon the PCOS model, we further explored the effects of DPP4 inhibition on the decidualization of endometrial stromal cells and changes in endometrial receptivity. Due to ovulatory dysfunction and infrequent menstruation, PCOS patients struggle to enter the luteal phase without external therapeutic intervention [11, 12]. Given this context, the assessment and investigation of endometrial receptivity defects in PCOS should commence from the proliferative phase and extend into the decidualization process [7, 8]. Although the impaired decidualization mechanisms by which PCOS affects endometrial receptivity remain unclear, it is associated with micro-level alterations such as oxidative stress and mitochondrial damage [69–72]. Our subsequent research demonstrated that the HD DHEA-induced endometrial stromal cells exhibited difficulties in decidualization capacity. Specifically, the invasion area of BeWo blastocyst-like cell spheres was smaller than that of normal decidualized T-HESCs. Importantly, T-HESCs treated with sitagliptin n exhibited restored decidualization, as evidenced by a larger invasion area compared to the untreated Dec-HD-DHEA-induced group.. These findings align with current research indicating that while physiological levels of DHEA can enhance decidualization, hyperandrogenism inhibits it in PCOS [73–76]. Based on these observations, our findings demonstrate that DPP4 inhibition effectively rescues the impaired decidualization capacity and restores functional endometrial receptivity in a hyperandrogenism-induced PCOS model. This provides compelling evidence that targeting the DPP4 pathway could represent a promising therapeutic strategy to overcome endometrial dysfunction in women with PCOS.

This study confirms that targeted modulation of DPP4 can effectively enhance endometrial receptivity in PCOS models, however, our findings should be considered alongside several limitations. First, as sitagliptin is primarily an antidiabetic drug, we cannot rule out that its beneficial effects on the endometrium are indirectly mediated through the amelioration of systemic insulin resistance in PCOS rats. Future studies using conditional knockout models or local intrauterine drug delivery could help isolate its direct effects. Second, given the inherent complexity of whole-animal studies, embryo transfer experiments would provide more definitive evidence for sitagliptin’s ability to improve functional receptivity and support implantation by better controlling for embryonic variables. Furthermore, the development of more physiologically relevant in vitro models is crucial. Future work should aim to simulate the hormonal sequential exposure (estrogen followed by estrogen plus progesterone) of the human menstrual cycle to establish a more robust and precise model of decidualization for PCOS research. Recent studies reported that serum DPP4 activity is significantly elevated in PCOS patients and is positively correlated with androgen levels [77–79]. In line with these observations, our preliminary evaluation of clinical endometrial specimens and reproductive outcomes further corroborates the above findings, showing improved endometrial receptivity and higher live birth rates following DPP4-targeted intervention. Collectively, this study provides multi-level evidence from molecular and functional assays to preliminary clinical data, supporting the role of DPP4 inhibition in ameliorating endometrial dysfunction in PCOS. While further validation is needed to address the current limitations, our work highlights the translational potential of targeting DPP4 to improve reproductive outcomes in this population.

## Conclusion

In summary, this study investigated the impact of targeted inhibition of DPP4 on endometrial receptivity in a HD DHEA-induced rat and cell model. Our results demonstrate that pharmacological inhibition of DPP4 significantly improved endometrial receptivity. DPP4 is not only a potential biomarker but also a key regulator of ferroptosis, with its expression levels negatively correlated with endometrial function. We found that suppressing ferroptosis significantly improved endometrial receptivity, highlighting the therapeutic potential of ferroptosis inhibition for enhancing fertility in PCOS. The mechanism by which DPP4 inhibition regulates ferroptosis provides key mechanistic insights into PCOS pathophysiology and suggests a novel therapeutic strategy. Future studies should explore the interactions between DPP4 and other biomarkers to develop more effective treatments.

## Supplementary Information

Additional file 1

## Data Availability

The data underlying this article will be shared on reasonable request to the corresponding author.
