# Supplementary material for: The mechanism study of targeting DPP4 in regulating ferroptosis and its influence on endometrial receptivity in PCOS
Source: Biol Sex Differ. 2025 Dec 23;16:107. doi: 10.1186/s13293-025-00786-5 (PMC12729179; doi:10.1186/s13293-025-00786-5)
Supplement: Supplementary file 1 — Additional file 1 [file 13293_2025_786_MOESM1_ESM.docx]

**Supplementary Figures**


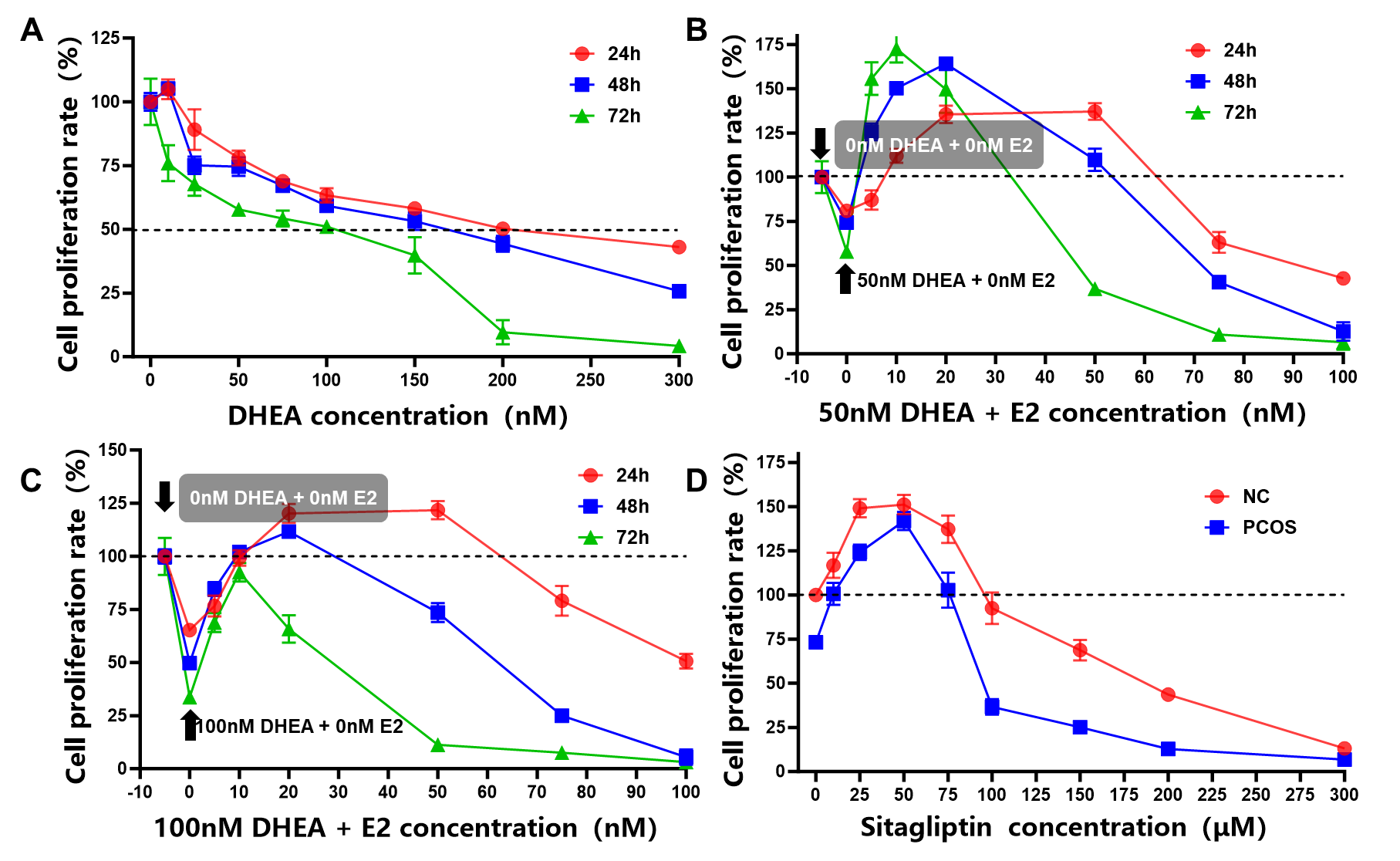


**Supplementary** **figure 1. CCK-8 experiment for screening optimal concentrations of various drugs.**

**A.** CCK-8 results of DHEA concentration in 24, 48, and 72h. **B.** CCK-8 results of E2 concentration in 50nM DHEA for the Control group. **C.** CCK-8 results of E2 concentration in 100nM DHT for the PCOS group. **D.** CCK-8 results of sitagliptin concentration in NC and PCOS group.

**Supplementary** **Figure 2. Establishment and verification of PCOS rats.**

**A.** Estrous cycle in NC, control, and PCOS group. **B.** Analysis of the estrous cycle in each group. **C.** The level of Sex hormone (testosterone, LH, FSH, and LH/FSH) in each group. **D.** The HE staining of ovaries in each group. **E.** The DPP4, GPX4, ER, and AR each group were determined by immunofluorescence and fluorescence MOD value analysis. **F.** The DPP4, GPX4, ER, and AR each group were determined by western blotting and analysis. Data are presented as mean ± SEM. All analysis were used Kruskal–Wallis test. ns, not significant. * *p*  < 0.05, ** *p*  < 0.01, *** *p* < 0.001, **** *p* < 0.0001.


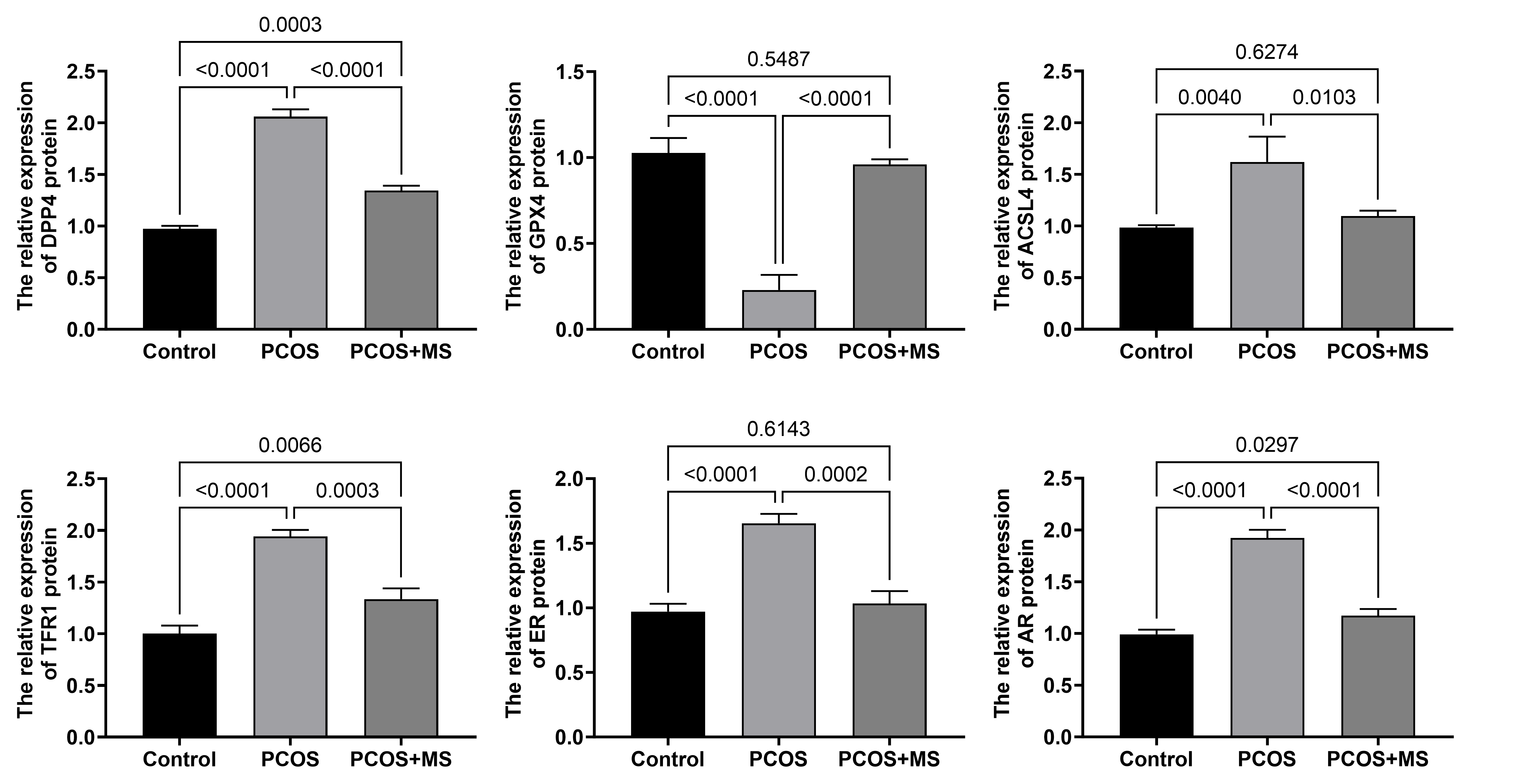


**Supplementary** **figure 3. Western blotting analysis in Figure 3**

The DPP4, GPX4, ACSL4, TFR1, ER, and AR each group determined by western blotting analysis (n=3), one-way ANOVA test. Data are presented as mean ± SEM.


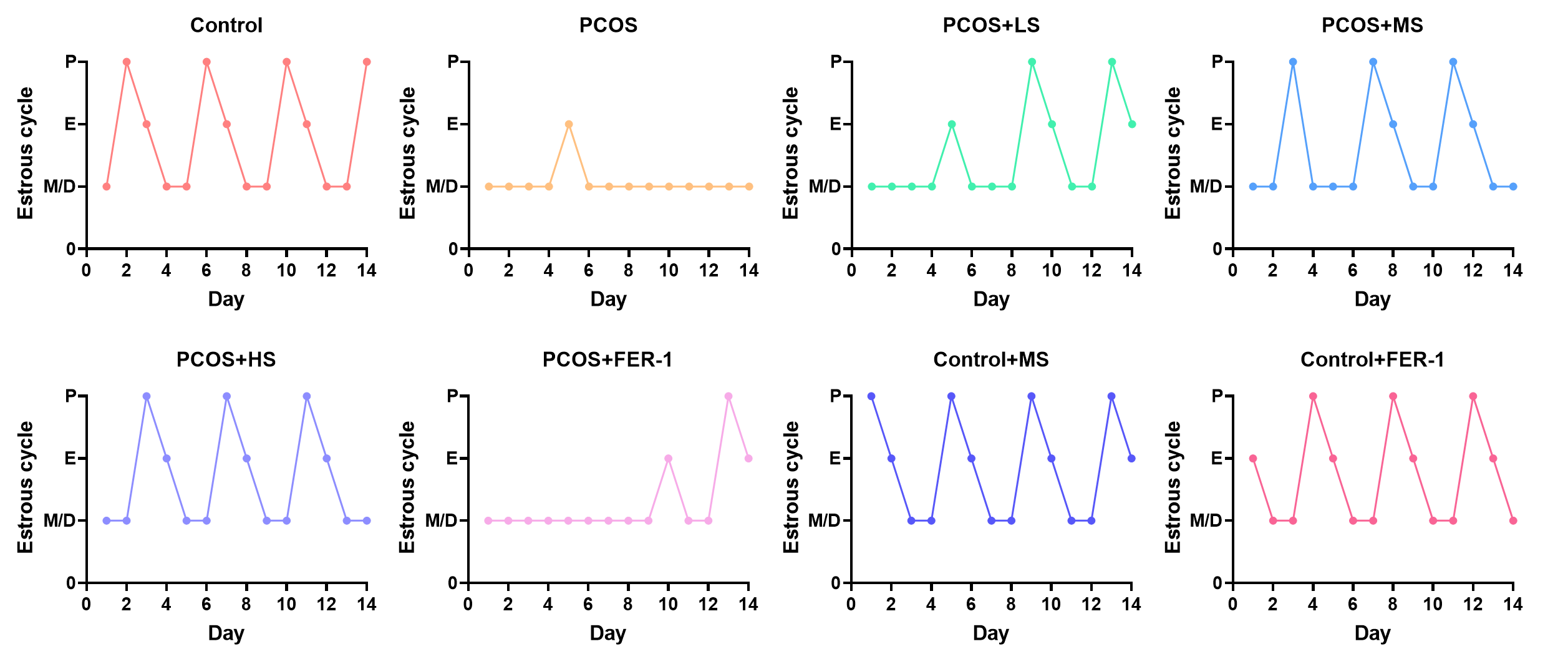


**Supplementary** **figure 4. Sitagliptin improved the disrupted estrous cycle of PCOS-like rats in each group.**


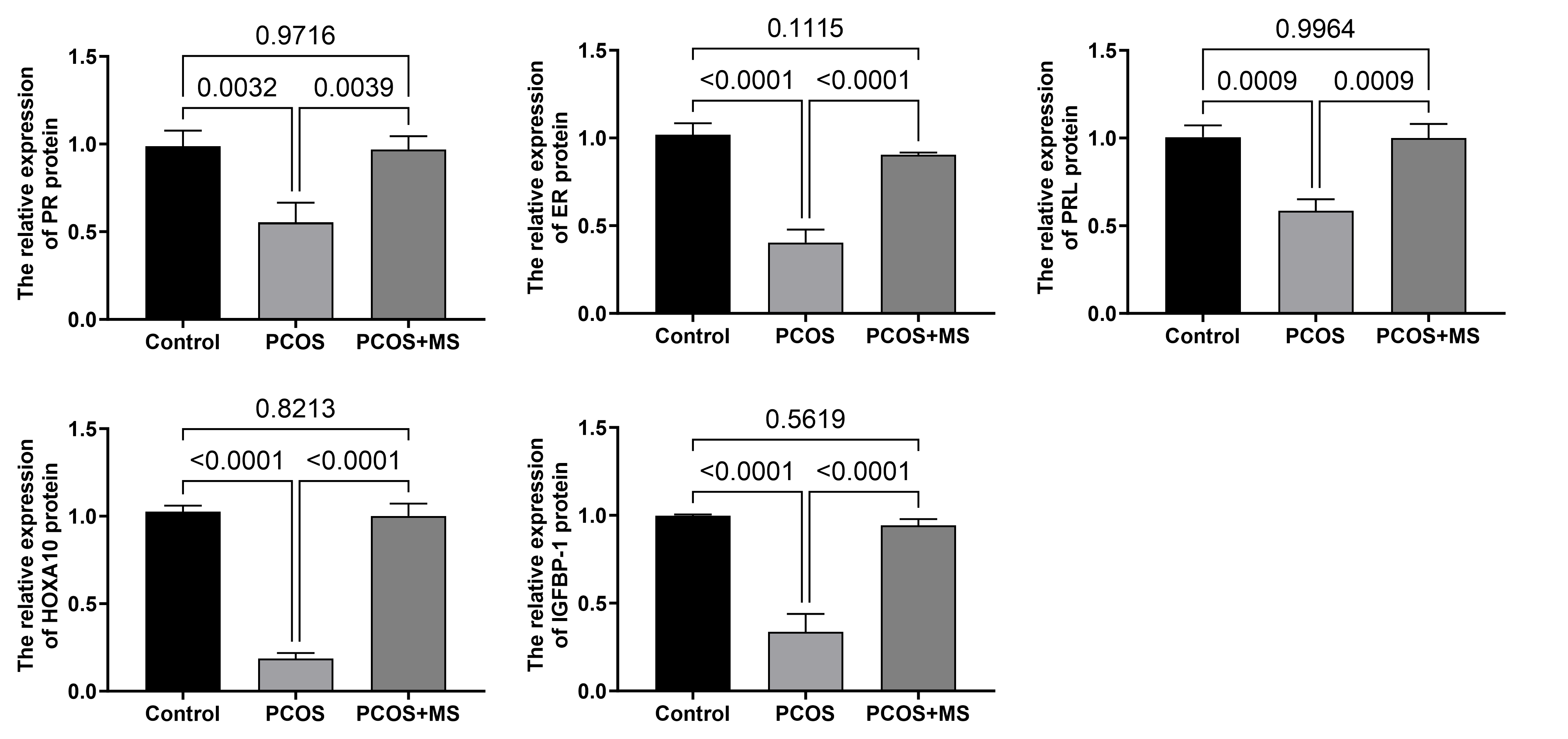


**Supplementary** **figure 5. Western blotting analysis in Figure 4**

The ER, PR, PRL, IGFBP-1, and HOXA10 each group determined by western blotting analysis (n=3), one-way ANOVA test. Data are presented as mean ± SEM.


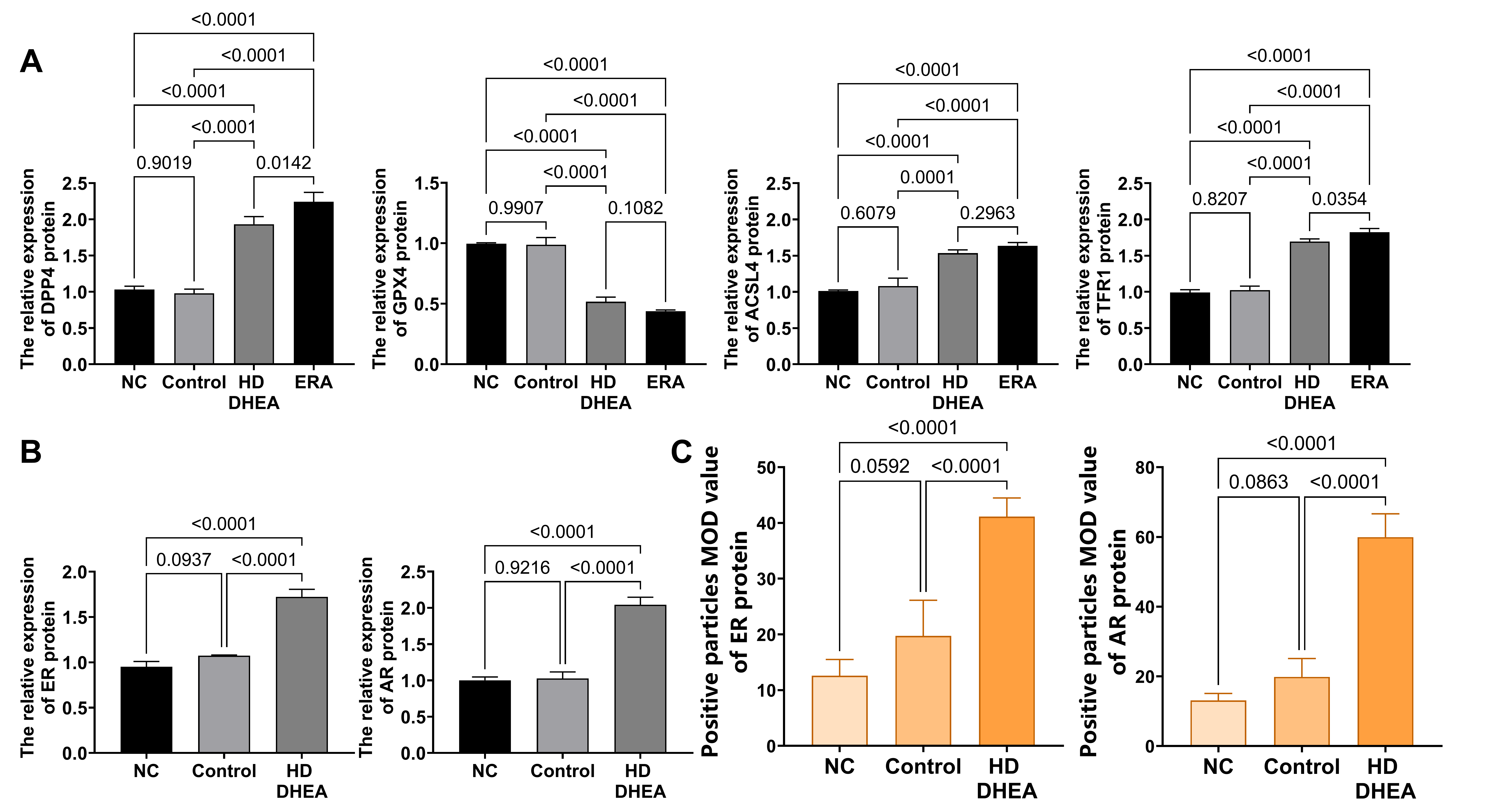


**Supplementary** **figure 6. Western blotting and** **immunohistochemistry analysis in Figure 5**

**A.** The DPP4, ACSL4, TFR1, and GPX4 each group determined by western blotting analysis (n=3), one-way ANOVA test. **B.** ER and AR each group determined by western blotting analysis (n=3), one-way ANOVA test. The ER and AR in each group were determined by immunohistochemistry and positive particles MOD value analysis (n=6), one-way ANOVA test. Data are presented as mean ± SEM.


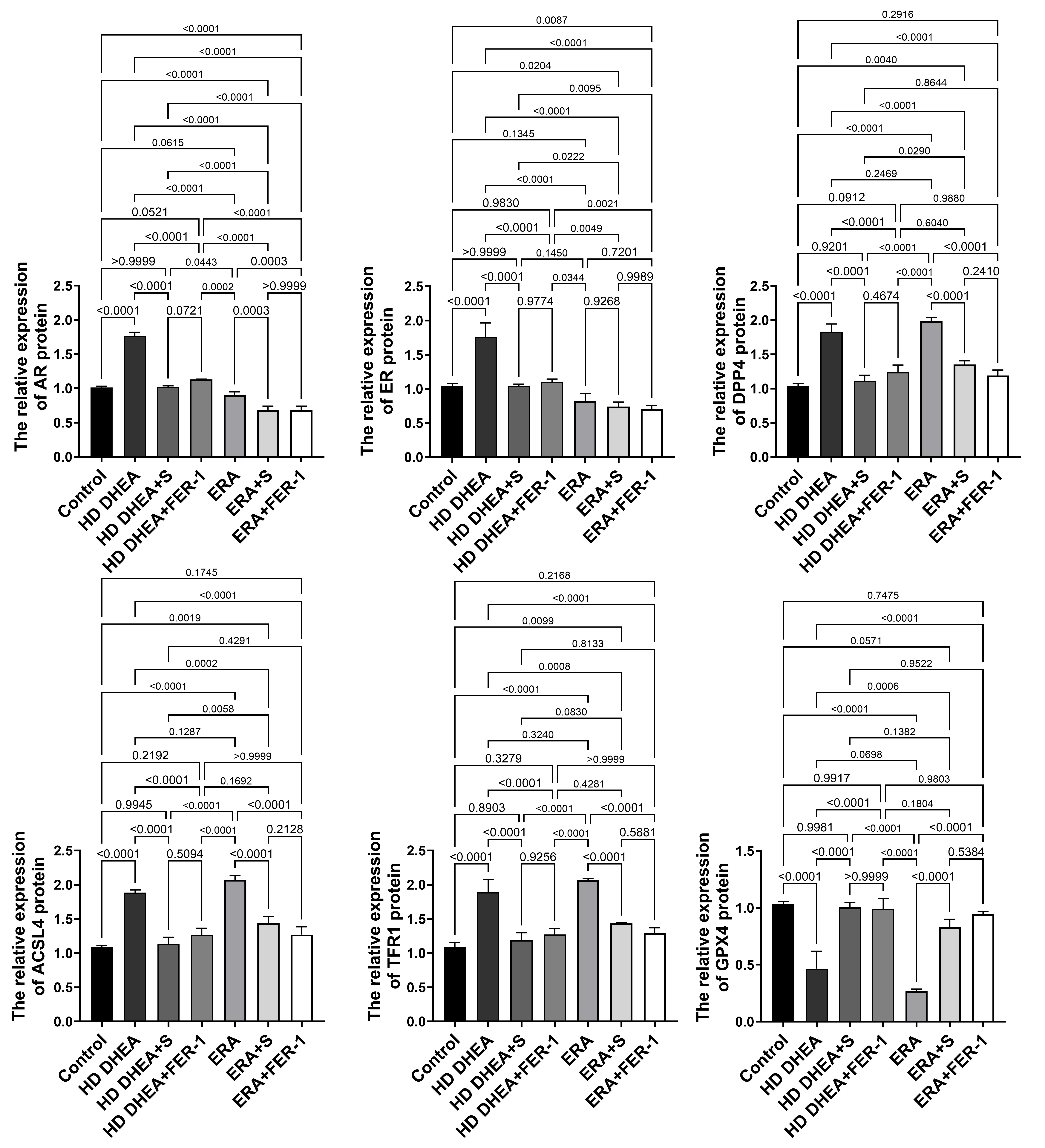


**Supplementary** **figure 7. Western blotting analysis in Figure 7**

The DPP4, GPX4, ACSL4, TFR1, ER, and AR each group were determined by western blotting analysis (n=3), one-way ANOVA test. Data are presented as mean ± SEM.


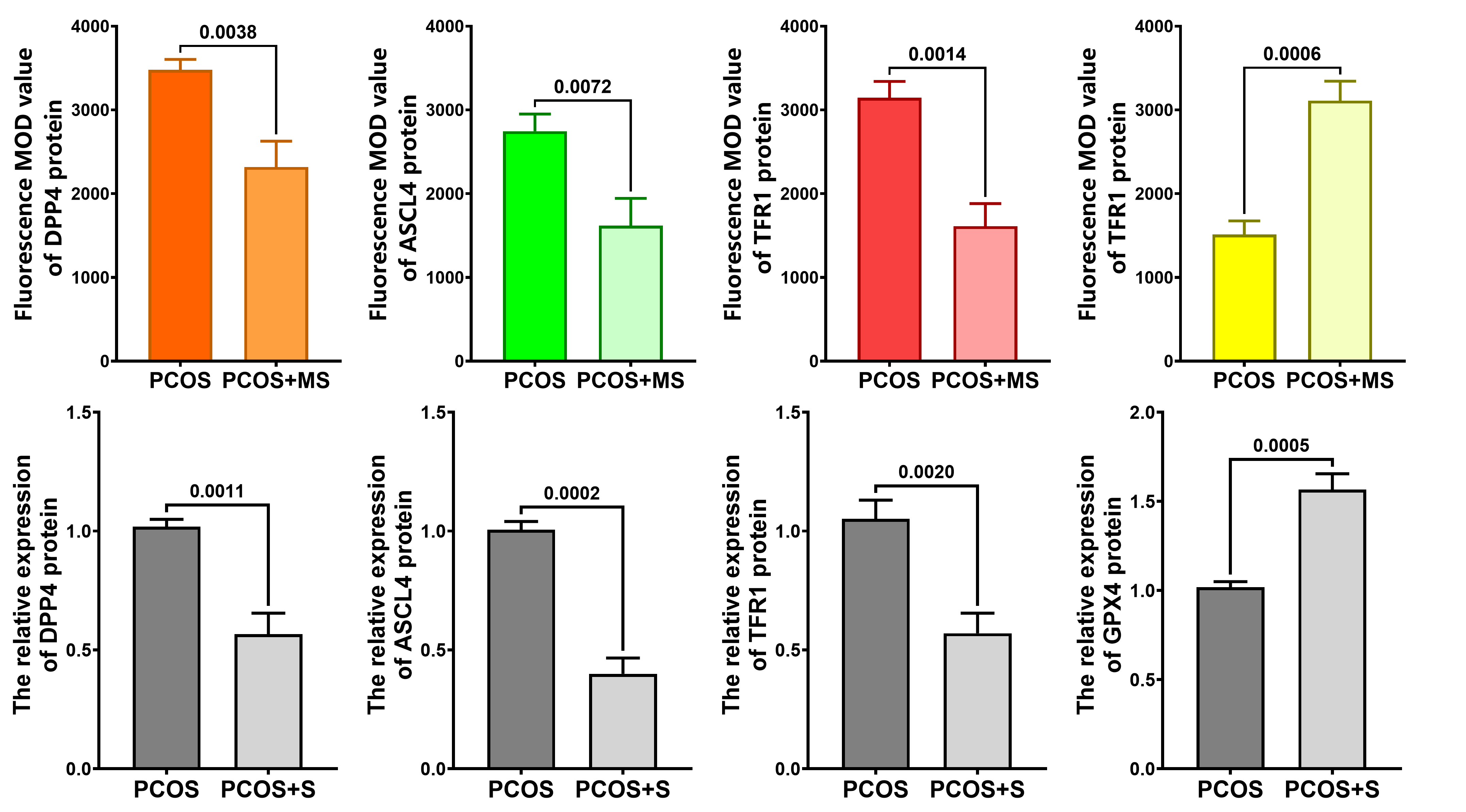


**Supplementary** **figure 8. Immunofluorescence and western blotting analysis in Figure 9.**

The DPP4, ACSL4, TFR1, and GPX4 each group were determined by immunofluorescence and western blotting analysis (n=6), t-test. Data are presented as mean ± SEM.
